# Optimizing water-efficient agriculture: evaluating the sustainability of soil management and irrigation synergies using fuzzy extent analysis

**DOI:** 10.1038/s41598-025-15426-6

**Published:** 2025-08-11

**Authors:** Rishikesh Sharma, Srinivas Rallapalli, Joe Magner

**Affiliations:** 1 Department of Civil Engineering, Birla Institute of Technology and Science, Pilani, Pilani Campus, Vidya Vihar, Pilani, 333031 Rajasthan India; 2 Department of Bioproducts and Biosystems Engineering, University of Minnesota, Twin cities, Minneapolis, USA

**Keywords:** Agriculture, Fuzy logic, Irrigation, Sustainability, Tillage, Hydrology, Civil engineering, Mathematics and computing

## Abstract

Sustainable agriculture demands the integration of optimized irrigation and soil tillage practices. Poor selection or mismatched combinations of these practices can lead to inefficient resource use, declining soil health, and reduced crop productivity. Despite extensive research on individual tillage and irrigation methods, limited studies explored their combined effects on multiple agricultural sustainability parameters. This gap underscores the need for a comprehensive assessment framework that can guide farmers and stakeholders in identifying optimal combinations for diverse agricultural objectives. This study employs a compounded fuzzy extent analysis to evaluate the cumulative impact of various soil tillage and irrigation methods on key agricultural parameters, including affordability, maximum yield, climate resilience, water usage, soil disruption, disease resistance, ease of operation, nutrient utilization, and crop diversification. The analysis compares individual practices and their combinations using comparative matrices to identify the most suitable options across all parameters. The fuzzy logic approach addresses data uncertainty by converting linguistic variables into triangular fuzzy numbers, enabling more accurate decision-making. The results indicate that Zero Tillage is the most effective tillage practice (score of 0.176), while Deficit Irrigation emerges as the most efficient irrigation method, scoring 0.144. The research suggests that integrating Zero-Tillage (ZT) with Deficit Irrigation (DI) is the most cost-effective agricultural practice. Additionally, combining No-Tillage (NT) with Surface Irrigation and Mulching (SIM) results in higher yields and improved water use efficiency. Furthermore, the synergy of No-Tillage (NT) and Drip Irrigation (DI) enhances crop resilience to climate change. These findings provide valuable insights for developing sustainable agricultural strategies that balance productivity, resource conservation, and environmental protection.

**Supplementary Information:**

The online version contains supplementary material available at 10.1038/s41598-025-15426-6.

## Introduction

 Sustainable agriculture requires the strategic integration of water management and soil tillage practices^1^. With growing concerns about water scarcity, soil degradation, and the need to increase agricultural output, finding the best combination of irrigation practices and tillage methods has become a pressing challenge. Efficient resource management is vital for improving crop productivity while conserving water and maintaining soil health. However, existing research has addressed these practices independently, limiting their real-world applicability as they didn’t explore how these two practices interact under different environmental and crop conditions^2^.

Soil tillage practices, such as Zero Tillage (ZT), No-Tillage (NT), Reduced Tillage (RT), Strip Mulching (SM), Conservation Tillage, Conventional Tillage with Broadcasting (CTB), Advanced Tillage (AT), Deep Tillage (DT), and Conventional Tillage (CT), play a crucial role in shaping soil health, crop yields, and environmental sustainability. Each method offers unique advantages and limitations, requiring careful selection based on site-specific conditions and crop requirements. Zero Tillage (ZT) minimizes soil disturbance, preserving structure and organic matter, but often necessitates increased herbicide use for weed control^3,4^. No-Tillage (NT) improves microbial diversity and moisture retention but may delay planting due to cooler soil temperatures. Reduced Tillage (RT) enhances soil structure and reduces costs but may not fully alleviate compaction^5^. Strip Mulching (SM) retains moisture and suppresses weeds but, if poorly managed, can attract pests. Conservation Tillage reduces erosion and fuel use but may increase herbicide dependency. Conventional Tillage with Broadcasting (CTB) benefits early-season crops but leads to significant erosion and moisture loss^6^. Advanced Tillage (AT) improves seedbed preparation but can degrade soil structure and raise fuel consumption^4^. Deep Tillage (DT) breaks compacted layers and enhances water infiltration, though excessive use may disrupt soil structure. Conventional Tillage (CT) is effective for weed control but causes long-term soil degradation and organic matter loss^6^.

Irrigation methods such as Sprinkler Irrigation (SI), Drip Irrigation (DI), Micro-sprinkler Irrigation (MI), Alternate Furrow Irrigation (Alt. FI), Partial Root-Zone Drying (PRD), Deficit Irrigation (Def. Irr.), Gated Pipe Irrigation (GPI), Surface Irrigation with Mulch (SIM), and Conventional Flood Irrigation (CFI) play a critical role in water management and crop production. Each approach offers unique benefits and challenges, depending on soil conditions, crop requirements, and water availability. Sprinkler Irrigation (SI) mimics rainfall, providing uniform water distribution across different soils, but it is energy-intensive and prone to evaporation losses^7,8^. Drip Irrigation (DI) delivers water directly to the root zone, improving efficiency and yields, though it demands high installation and maintenance costs^9^. Micro-sprinkler Irrigation (MI) is suitable for orchards, offering localized water application with minimal evaporation, but requires filtration to prevent clogging^10^. Alternate Furrow Irrigation (Alt. FI) reduces water usage by nearly 50% but may result in uneven moisture distribution. Partial Root-Zone Drying (PRD) enhances drought tolerance and water efficiency by alternating irrigation to different root zones, yet mismanagement can cause water stress^11^. Deficit Irrigation (Def. Irr.) intentionally limits water during specific growth stages to save water, requiring precision to avoid yield losses. Gated Pipe Irrigation (GPI) improves uniformity and reduces labor compared to traditional furrow irrigation, though initial costs are high. Surface Irrigation with Mulch (SIM) retains moisture and reduces evaporation, but its labor-intensive nature can be a limitation^12^. Conventional Flood Irrigation (CFI), one of the oldest methods, is simple and cost-effective but highly inefficient, leading to water loss, soil erosion, and nutrient leaching^9^.

Presently, drought and water stress rank among the most significant factors impacting crop yield and quality^13^. On the other hand, soil tillage significantly affects soil, air, and water quality by influencing greenhouse gas exchange and the movement of nutrients and pesticides^14^. Thus, it is evident that irrigation and soil tillage methods significantly impact various aspects of agriculture, including sustainability, water conservation, crop yield, soil water content, carbon cycle, organic carbon, greenhouse gas emissions, carbon sequestration, surface runoff, and soil physical properties. Montazar et al.^15^ and Kumar et al.^16^ examined soil tillage techniques like Conventional Tillage (CT), Zero tillage (ZT), Reduced tillage (RT), and Conventional Tillage with Bed Planting (CTB) for their impact on productivity, yield, and profitability, but does not consider irrigation practices for these parameters. Aula et al.^17^ investigated common tillage practices for yield predictions but failed to address data inconsistencies related to Moldboard Plow (MP) and limits its focus to yield production. Additionally, Kumar et al.^16^ explores multiple irrigation practices and soil tillage methods, but narrows its focus to crop productivity, soil health, and water-use efficiency. Saglam et al.^18^ investigates the effects of different tillage practices, including Conventional Tillage (CT), Reduced Tillage (RT), and Zero Tillage (ZT), on soil compaction and crop characteristics. While the study focuses on soil compaction, moisture levels, and wheat production, it lacks a comprehensive analysis of other crucial aspects such as biodiversity, the sequestration of soil organic carbon, the efficiency of water usage and a few other parameters. Tobiašová et al.^19^ studied reduced and conventional tillage are compared, the study does not include no-till systems, which are critical in discussions of sustainable agriculture and carbon sequestration.

Evidently, most of the studies independently studied the impact of soil tillage and irrigation practices that too on a small set of parameters without considering their uncertain and inconsistent nature. Consequently, evaluating the combined effects of diverse soil tillage and irrigation techniques based on comprehensive set of uncertain parameters is crucial. The combination of irrigation and tillage practices is far from straightforward. Conventional tillage, while widely adopted, is known to accelerate soil erosion and reduce organic matter, ultimately harming long-term soil fertility. On the other hand, conservation tillage, when paired with modern irrigation techniques such as drip or deficit irrigation, has shown potential to reduce water use and improve soil structure. Despite the clear need for a holistic approach, the literature often focuses on the effects of irrigation and tillage in isolation, neglecting their combined influence on soil moisture dynamics, crop performance, and resource use efficiency. There is a critical need for research that investigates how these practices can be paired together to meet both environmental and agricultural goals. As climate change intensifies and resources become increasingly limited, identifying synergistic combinations of tillage and irrigation strategies is essential. This paper aims to bridge that knowledge gap by examining how integrated approaches can enhance water-use efficiency, promote sustainable soil management, and improve overall agricultural resilience. Understanding these interactions will provide valuable insights for developing site-specific solutions that address both current challenges and future uncertainties in agricultural systems.

Fuzzy logic has emerged as a crucial tool for assessing multiple alternatives, such as irrigation and soil tillage practices, while addressing uncertainties in these alternatives Srinivas^20^ and Champaneri et al.^21^ applied the fuzzy analytic hierarchy process (FAHP) to evaluate sustainability in leisure agriculture, incorporating environmental effects, resource distribution, and operational productivity into the assessment. The study demonstrated how fuzzy-based methods offer practical guidance for decision-makers and improve sustainability evaluations. Hayat et al.^22^ employed fuzzy-set qualitative comparative analysis (fsQCA) to analyze factors influencing conservation agriculture adoption, such as risk tolerance, ecological awareness, and financial incentives. By measuring adoption levels beyond binary classifications, the research provided a more comprehensive understanding of farmer behavior. Keshavarzi et al.^23^ combined FAHP with parametric techniques to assess soil fertility for wheat and maize^24^. The study integrated soil characteristics like organic matter, texture, pH, and nutrient levels into a fuzzy decision framework, offering adaptable assessments across varying climatic and soil conditions. In precision agriculture, Nanavaty et al.^25^ and Sinha et al.^26^ investigated fuzzy logic techniques for disease detection and management using fuzzy membership functions and rule-based systems, demonstrating their utility in crop monitoring and automated disease control. Similarly, Aggarwal et al.^27^ showcased the effectiveness of fuzzy-based approaches for improving crop health management through case studies, further highlighting its potential in precision agriculture.

This research employs a fuzzy extent analysis to simultaneously evaluate the soil tillage and irrigation practices based on nine prominent factors namely affordability, maximum yield, climate-resilient crops, reduced water usage, minimal soil disruption, disease resistance, ease of operation, optimized nutrient utilization, and promotion of crop diversification. Fuzzy extent analysis allows for the conversion of linguistic variables into triangular fuzzy numbers, which effectively addresses data inconsistencies. This assessment not only examines and contrasts cultivation and water management techniques for individual parameters using comparative matrices but also analyzes combinations of these approaches for all parameters. The objectives of this study are to: (1) prioritize the criteria for evaluating soil tillage and irrigation practices, (2) determine the best soil tillage and irrigation techniques based on various evaluation criteria, and (3) derive the best combinations of tillage and irrigation methods. This approach allows farmers or stakeholders to select a mix of tillage and irrigation techniques that align with their objectives, such as cost-effectiveness or maximizing crop yield.

## Methodology

### Superiority of the proposed method

Fuzzy Extent Analysis (FEA), a form of the Fuzzy Analytic Hierarchy Process (FAHP), was introduced in 1996 to enhance decision-making in situations marked by ambiguity and uncertainty. This approach expands the conventional AHP model by employing triangular fuzzy numbers to depict subjective pairwise comparisons, thereby facilitating a more refined interpretation of expert opinions when precise judgments are impractical. The technique calculates a possibility degree for one alternative’s dominance over another, allowing for the derivation of normalized priority weights without the need for consistency checks, which are often cumbersome in the traditional AHP.

Fuzzy extent analysis has shown strong applicability in similar research areas. For example, Keshavarzi et al.^23^ employed fuzzy AHP for assessing soil fertility in wheat and maize cultivation in Iran, taking into account nutrient profiles and soil characteristics under uncertain conditions. Likewise, Hayat et al.^22^ used fuzzy-set Qualitative Comparative Analysis (fsQCA) to investigate farmer adoption behaviour for conservation agriculture, focusing on non-binary adoption patterns and expert-based reasoning. Beyond the core fuzzy MCDM literature, numerous recent applications underscore the soundness of this methodological approach. For instance, Hosini^28^ utilized fuzzy AHP to assess the suitability of sprinkler irrigation, showing greater sensitivity compared to conventional parametric techniques. In a similar vein, Tuncel & Gunturk^29^ integrated fuzzy TOPSIS with game theory to assist in selecting agricultural land amidst uncertain environmental and social conditions. Abuzaid et al.^30^ conducted a spatially explicit analysis that merged GIS with fuzzy logic and AHP for crop suitability mapping, illustrating the method’s replicability^24^. Rouyendegh & Savalan^31^ introduced a hybrid fuzzy MCDM model that merges Buckley’s fuzzy AHP with fuzzy TOPSIS to assess agricultural production methods amidst uncertainty. This model incorporates expert hesitation and linguistic evaluations to comprehensively rank sustainable options based on satisfaction, economic, and environmental criteria.

Unlike the traditional AHP, which relies on precise judgments and assumes consistent preferences, fuzzy extent analysis (FEA) offers a more adaptable and realistic approach to managing the uncertainty and vagueness often present in expert evaluations. Although classical AHP is widely utilized, research highlights its shortcomings. For example, Halder et al.^32^ conducted a comparative study of fuzzy AHP and classical AHP for selecting groundwater recharge sites. Their findings indicated that classical AHP struggled to handle subjective uncertainty in expert inputs, whereas fuzzy AHP, using Chang’s method, enhanced decision stability and gained greater stakeholder acceptance. In their evaluation of irrigation modernization strategies, Sun et al.^33^ noted that traditional AHP required consistency checks and was sensitive to minor judgment variations, resulting in inconsistent priority vectors. Conversely, Fuzzy Chang’s method effectively managed ambiguity and ensured reliable priority derivation. Lord et al.^34^ applied fuzzy AHP to prioritize irrigation schemes and discovered that the fuzzy extent analysis method bypassed the computational demands of Saaty’s consistency ratio, making it more scalable for large sets of alternatives. Kumar & Pant^35^ observed in their assessment of sustainable agriculture indicators in semi-arid regions that classical AHP was insufficient for capturing stakeholder hesitation, while fuzzy extent analysis provided richer linguistic expression and robustness in final rankings.

These examples demonstrate the method’s robustness and interdisciplinary adaptability, further supporting its relevance in our study. Therefore, the choice of fuzzy extent analysis is based not only on its methodological appropriateness but also on its proven effectiveness in agriculture, irrigation management, soil evaluation, and expert-driven prioritization—all of which closely align with the goals and constraints of the current research.

**Fig. 1:**
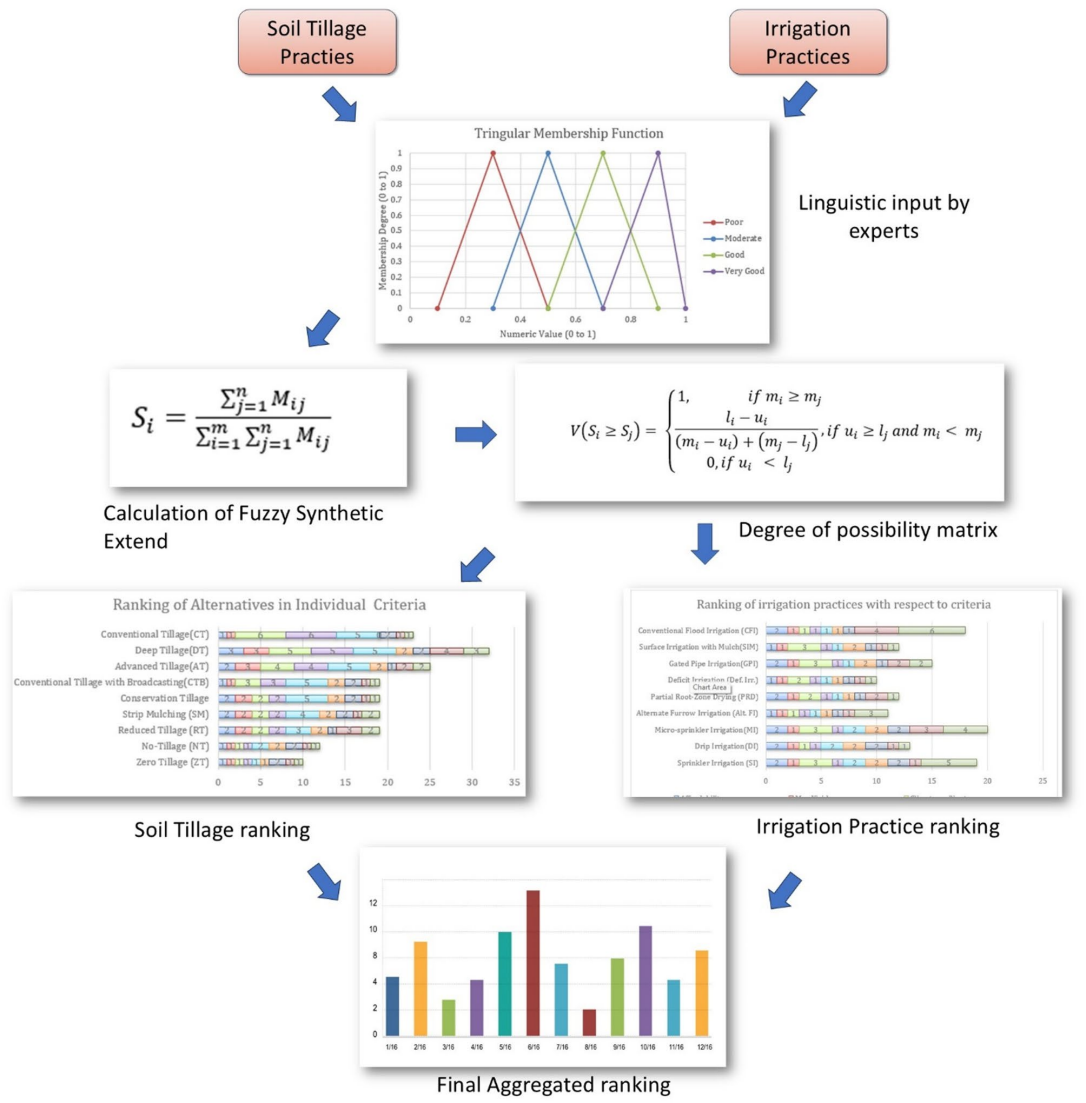
Schematic procedure adopted in the study.

The methodology integrates fuzzy logic to rank irrigation and soil tillage practices for optimizing agricultural performance. Expert opinions are collected as linguistic inputs, which are converted into triangular fuzzy numbers. Fuzzy synthetic extent analysis is used to calculate the degree of possibility matrix and rank alternatives. Individual rankings for soil tillage and irrigation practices are derived based on multiple criteria such as crop diversification, nutrient optimization, water conservation, and yield maximization. Finally, aggregated rankings are developed to identify the best combination of practices, ensuring a sustainable and efficient agricultural strategy for the region. The schematic methodology employed in this research is illustrated in Fig. 1.

### Study area

**Fig. 2:**
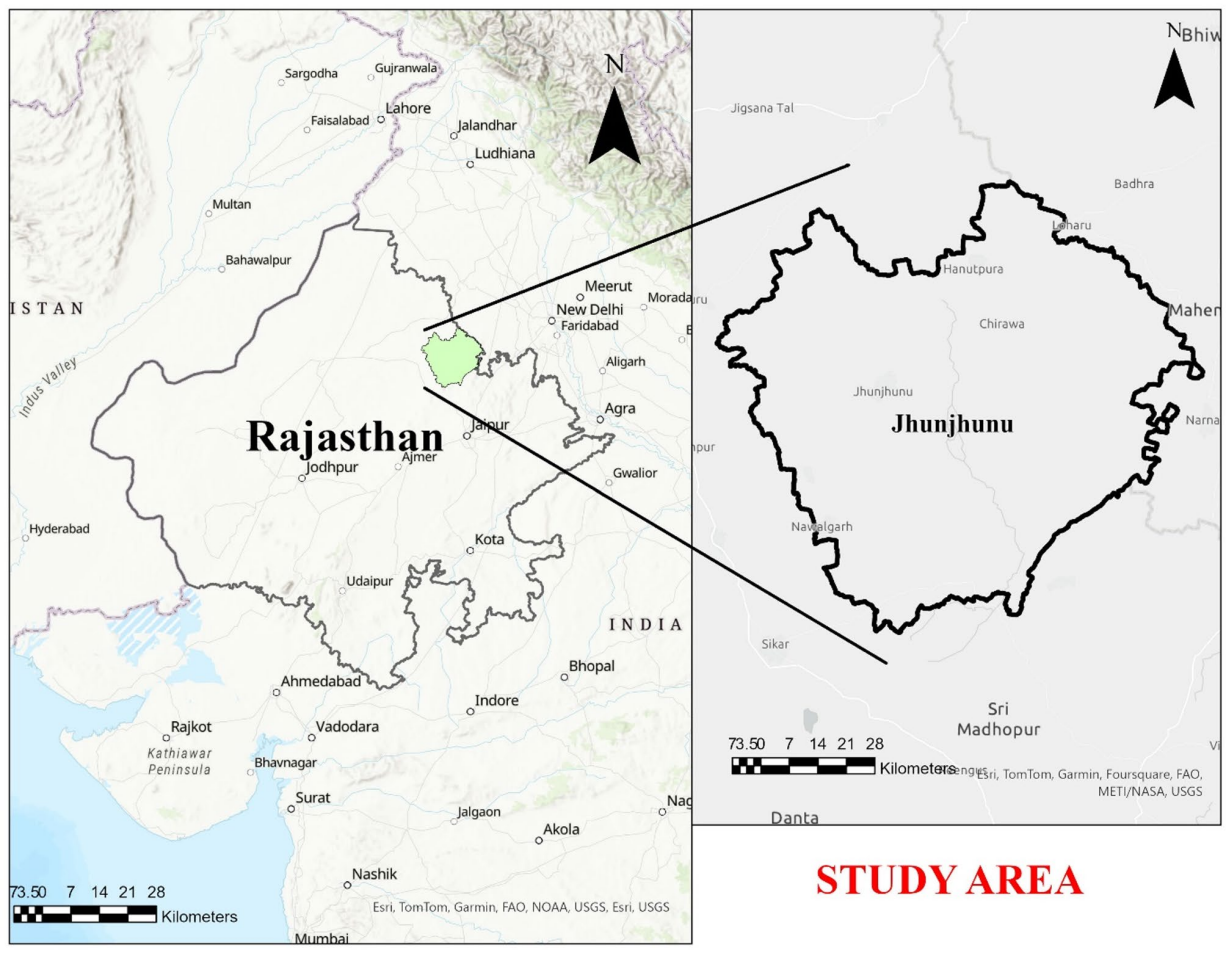
Representing Jhunjhunu district of Rajasthan (created using ArcGIS Version 10.9)^36^.

The research focuses on agricultural regions of Jhunjhunu, a district in Rajasthan (India), as its study area, Fig. 2^36^. Historically, farmers in this region have employed traditional tillage methods, which involve extensive plowing and repeated soil turning for seedbed preparation. However, these approaches often result in moisture depletion and heightened soil erosion. In response to these issues, a transition towards conservation tillage techniques has emerged. Methods such as zero tillage and reduced tillage, which minimize soil disturbance, have been adopted to improve moisture retention and decrease erosion^37^. Furthermore, practices like strip mulching and deep tillage are implemented to enhance water infiltration and nutrient absorption, particularly benefiting the rainfed crops common in the area. The implementation of these sustainable tillage practices is essential for preserving soil health and sustaining agricultural productivity in Rajasthan’s arid regions. Sprinkler Irrigation (SI) is a widely used irrigation method in Jhunjhunu^38^. According to the District Irrigation Plan, Jhunjhunu^39^, drip irrigation has gained popularity for horticultural and high value crops due to water scarcity, offering precise water delivery and reducing waste.

Jhunjhunu faces significant challenges due to water scarcity and improper soil tillage practices, which severely affect agricultural productivity. Limited water availability, combined with suboptimal tillage methods, leads to soil degradation, reduced water retention, and declining crop yields. Farmers often struggle to balance water conservation with efficient land preparation. Hence, there is an urgent need for a synergistic approach that integrates sustainable irrigation practices with suitable tillage techniques. This study aims to address these challenges and identify the best combination of irrigation and tillage practices to optimize water use and enhance agricultural sustainability in this semi-arid region.

### Data preparation

The research analyzed several secondary literatures^17,40,41^ to identify nine essential irrigation activities under the guidance of ten experts from academia, scientific institutions, industry, irrigation and agriculture department, water resources department. Sections A, B, and C of the supplementary material include the questionnaire, details about the experts, weight calculations (Table C.1, C.2) and criteria for parameter election. The research established nine critical criteria for assessing various irrigation and soil tillage methods (alternatives). These criteria, irrigation and soil tillage practices are presented in Table 1. The research also examined the relative weighted significance (score) of each criteria, as determined by experts.

**Table 1:** Soil tillage and irrigation practices and decision criteria.

Sr No.	Criteria	Soil Tillage Practices	Irrigation Practices
**1.**	Affordable	Zero Tillage (ZT)	Sprinkler Irrigation (SI)
**2.**	Max Yield	No-Tillage (NT)	Drip Irrigation (DI)
**3.**	Climate Resilient	Reduced Tillage (RT)	Micro-sprinkler Irrigation (MI)
**4.**	Water Less Consumption	Strip Mulching (SM)	Alternate Furrow Irrigation (Alt. FI)
**5.**	Less Soil Disturbed	Conservation Tillage	Partial Root-Zone Drying (PRD)
**6.**	Disease Resistance	Conventional Tillage with Broadcasting (CTB)	Deficit Irrigation (Def. Irr.)
**7.**	Easy Operation	Advanced Tillage (AT)	Gated Pipe Irrigation (GPI)
**8.**	Optimized Nutrient	Deep Tillage (DT)	Surface Irrigation with Mulch (SIM)
**9.**	Promoting Crop Diversification	Conventional Tillage (CT)	Conventional Flood Irrigation (CFI)

### Aggregating the expert opinions

**Fig. 3:**
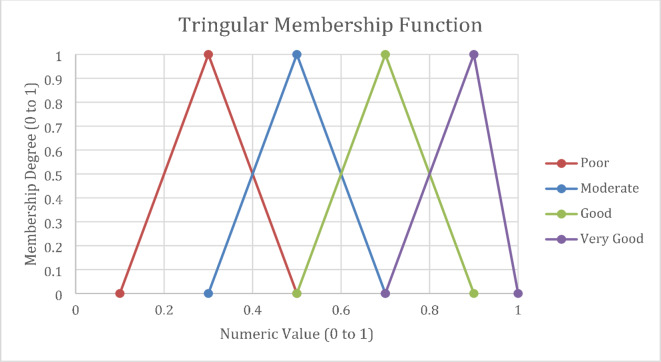
Membership functions and corresponding linguistic ratings.

The research incorporated expert evaluations to determine the extent to which specific practices met the established criteria. Fuzzy extent analysis was applied to various alternatives for both irrigation and soil tillage techniques. Additionally, the study accounted for the importance assigned to each criterion by the experts through weights. Figure 3 presents the linguistic inputs with triangular membership functions, Tables 2 and 3 present the respective matrix inputs.

Tables 2 and 3 present the linguistic ratings derived for soil tillage and irrigation practices through experts and secondary literature, along with the weights assigned to each criterion. The linguistic inputs indicate how well each alternative or practice is rated for specific criteria (Table 2). For instance, Sprinkler Irrigation (SI) is considered moderately affordable. Likewise, Conventional Flood Irrigation (CFI) is rated poorly in terms of climate resilience, suggesting that using CFI has a low likelihood of producing climate-resilient crops. In a similar vein, Zero Tillage (ZT) is rated as good, indicating that this practice is highly affordable (Table 3).

**Table 2:** Linguistic ratings of irrigation practices with respect to decision criteria.

Criteria	Weights	Sprinkler Irrigation (SI)	Drip Irrigation	Micro-sprinkler Irrigation	Alternate Furrow Irrigation (Alt. FI)	Partial Root-Zone Drying (PRD)	Deficit Irrigation (DI)	Gated Pipe Irrigation	Surface Irrigation with Mulch	Conventional Flood Irrigation (CFI)
Affordable	0.18	M	M	M	G	M	G	M	G	G
Max Yield	0.16	G	VG	M	M	M	M	M	M	M
Climate Resilient	0.11	M	VG	M	M	G	M	M	M	P
Water Less Consumption	0.13	G	VG	G	G	G	G	G	G	P
Less Soil Disturbed	0.14	M	G	G	G	G	G	G	G	P
Disease Resistance	0.1	M	G	M	M	M	M	M	M	M
Easy Operation	0.08	M	M	M	G	M	G	M	M	M
Optimized Nutrient	0.06	M	VG	G	M	G	M	M	M	P
Promoting Crop Diversification	0.1	M	M	M	M	M	M	M	M	P

**Table 3:** Linguistic ratings of tillage practices with respect to decision criteria.

Criteria	Weights	Zero Tillage (ZT)	No-Tillage (NT)	Reduced Tillage (RT)	Stubble Mulch Tillage (SM)	Conservation Tillage	Conventional Tillage with Bed Planting (CTB)	Agronomic Tillage	Deep Tillage	Conventional Tillage (CT)
Affordable	0.18	G	G	M	M	M	G	M	P	G
Max Yield	0.16	VG	VG	G	G	G	G	M	M	M
Climate Resilient	0.11	VG	VG	G	G	G	M	M	M	P
Water Less Consumption	0.13	VG	VG	G	G	G	M	M	M	P
Less Soil Disturbed	0.14	VG	VG	G	G	G	M	M	M	P
Disease Resistance	0.1	G	G	M	M	M	M	M	M	M
Easy Operation	0.08	M	M	G	M	M	M	G	M	M
Optimized Nutrient	0.06	G	G	M	G	G	M	M	M	P
Promoting Crop Diversification	0.1	G	G	M	M	M	M	M	M	P

The linguistic ratings are subsequently transformed into triangular fuzzy numbers, with each linguistic value assigned a specific triangular fuzzy number. For instance, the value “good” is represented by (0.5,0.7,0.9) in Table 4 for Zero Tillage (ZT) under the criterion- affordability. Similarly, conventional tillage (CT) is assigned (0.3,0.5,0.7) for “poor” in the climate resilience category. This conversion is carried out to facilitate the subsequent fuzzy extent analysis process.

**Table 4:** Fuzzification of linguistic ratings using triangular fuzzy numbers for soil practices.

Criteria	Zero Tillage (ZT)	No-Tillage (NT)	Reduced Tillage (RT)	Stubble Mulch Tillage (SM)	Conservation Tillage	Conventional Tillage with Bed Planting (CTB)	Agronomic Tillage	Deep Tillage	Conventional Tillage (CT)
**Affordable**	(0.5, 0.7, 0.9)	(0.5, 0.7, 0.9)	(0.3, 0.5, 0.7)	(0.3, 0.5, 0.7)	(0.3, 0.5, 0.7)	(0.5, 0.7, 0.9)	(0.3, 0.5, 0.7)	(0.1, 0.3, 0.5)	(0.5, 0.7, 0.9)
**Max Yield**	(0.7, 0.9, 1.0)	(0.7, 0.9, 1.0)	(0.5, 0.7, 0.9)	(0.5, 0.7, 0.9)	(0.5, 0.7, 0.9)	(0.5, 0.7, 0.9)	(0.3, 0.5, 0.7)	(0.3, 0.5, 0.7)	(0.3, 0.5, 0.7)
**Climate Resilient**	(0.7, 0.9, 1.0)	(0.7, 0.9, 1.0)	(0.5, 0.7, 0.9)	(0.5, 0.7, 0.9)	(0.5, 0.7, 0.9)	(0.3, 0.5, 0.7)	(0.3, 0.5, 0.7)	(0.3, 0.5, 0.7)	(0.1, 0.3, 0.5)
**Water Less Consumption**	(0.7, 0.9, 1.0)	(0.7, 0.9, 1.0)	(0.5, 0.7, 0.9)	(0.5, 0.7, 0.9)	(0.5, 0.7, 0.9)	(0.3, 0.5, 0.7)	(0.3, 0.5, 0.7)	(0.3, 0.5, 0.7)	(0.1, 0.3, 0.5)
**Less Soil Disturbed**	(0.7, 0.9, 1.0)	(0.7, 0.9, 1.0)	(0.5, 0.7, 0.9)	(0.5, 0.7, 0.9)	(0.5, 0.7, 0.9)	(0.3, 0.5, 0.7)	(0.3, 0.5, 0.7)	(0.3, 0.5, 0.7)	(0.1, 0.3, 0.5)
**Disease Resistance**	(0.5, 0.7, 0.9)	(0.5, 0.7, 0.9)	(0.3, 0.5, 0.7)	(0.3, 0.5, 0.7)	(0.3, 0.5, 0.7)	(0.3, 0.5, 0.7)	(0.3, 0.5, 0.7)	(0.3, 0.5, 0.7)	(0.3, 0.5, 0.7)
**Easy Operation**	(0.3, 0.5, 0.7)	(0.3, 0.5, 0.7)	(0.5, 0.7, 0.9)	(0.3, 0.5, 0.7)	(0.3, 0.5, 0.7)	(0.3, 0.5, 0.7)	(0.5, 0.7, 0.9)	(0.3, 0.5, 0.7)	(0.3, 0.5, 0.7)
**Optimized Nutrient**	(0.5, 0.7, 0.9)	(0.5, 0.7, 0.9)	(0.3, 0.5, 0.7)	(0.5, 0.7, 0.9)	(0.5, 0.7, 0.9)	(0.3, 0.5, 0.7)	(0.3, 0.5, 0.7)	(0.3, 0.5, 0.7)	(0.1, 0.3, 0.5)
**Promoting Crop Diversification**	(0.5, 0.7, 0.9)	(0.5, 0.7, 0.9)	(0.3, 0.5, 0.7)	(0.3, 0.5, 0.7)	(0.3, 0.5, 0.7)	(0.3, 0.5, 0.7)	(0.3, 0.5, 0.7)	(0.3, 0.5, 0.7)	(0.1, 0.3, 0.5)

### Fuzzy extent analysis

#### Calculation of fuzzy synthetic extent

The fuzzy synthetic extent technique plays a crucial role in the fuzzy analytic hierarchy process (FAHP), effectively handling ambiguity and subjectivity in complex decision-making scenarios by consolidating pairwise comparisons into comprehensive fuzzy values. This methodology enables systematic ranking and prioritization of options in situations characterized by vagueness or imprecision. Contemporary research has showcased its adaptability across numerous disciplines. For example, Jain et al.^42^ utilized this approach in the automotive sector for vendor selection, combining FAHP with TOPSIS to determine criteria weights and address fuzziness in expert assessments. In a similar vein, Kumar et al.^40^ implemented the fuzzy synthetic extent technique to assess renewable energy technologies, tackling sustainability issues by incorporating both qualitative and quantitative factors. These implementations highlight the method’s wide-ranging applicability and dependability in addressing intricate decision-making challenges across various fields. The triangular fuzzy numbers from Table 4 are further used to calculate fuzzy synthetic analysis using Eqs. (1–3).

Each pairwise comparison is expressed as a Triangular Fuzzy Number (TFN): $\:(l,m,u)$, where:

- $\:l$: Lower bound (minimum value).
- $\:m$: Middle value (most likely).
- $\:u$: Upper bound (maximum value).

1 $$\:{S}_{i}=\frac{{\sum\:}_{j=1}^{n}{M}_{ij}}{{\sum\:}_{i=1}^{m}{\sum\:}_{j=1}^{n}{M}_{ij}}$$

$\:Mij=\left({l}_{ij},{m}_{ij},{u}_{ij}\right)\:$ The fuzzy comparison value for alternative $\:i$ and criterion $\:j.$.

$\:{\sum\:}_{j=1}^{n}{M}_{ij}$ : Aggregation of all fuzzy values for alternative $\:i$ across $\:n$ criteria.

$\:{\sum\:}_{i=1}^{m}{\sum\:}_{j=1}^{n}{M}_{ij}$ : Total aggregation of all fuzzy values across all alternatives and criteria.

#### Fuzzy arithmetic calculations

The addition of Triangular Fuzzy Numbers (TFNs) adheres to the concept that combining fuzzy numbers should preserve their triangular form, thus maintaining the range of uncertainty. For a group of TFNs, the addition formula illustrates that the fuzzy sum is calculated by independently adding the corresponding lower, middle, and upper values. This feature is particularly useful in fuzzy extent analysis, which involves aggregating evaluations across multiple fuzzy numbers. The addition of TFNs is performed using Eq. (2).

2 $$\:\sum\:_{k=1}^{p}({l}_{k},\:{m}_{k},{u}_{k}):\:\left(\sum\:_{k=1}^{p}{l}_{k},\sum\:_{k=1}^{p}{m}_{k},\sum\:_{k=1}^{p}{u}_{k}\right)$$

When dividing TFNs element by element, the resulting fuzzy number maintains a consistent range of uncertainty and proportional relationships. This division technique is crucial for normalizing fuzzy synthetic values, which allows for comparison across different criteria in fuzzy decision-making frameworks. The process of dividing TFNs is carried out using Eq. (3). Further the normalization of fuzzy synthetic values takes place to ensure comparability across criteria.

3 $$\:\frac{\left({l}_{1},{m}_{1},{u}_{1}\right)}{\left({l}_{2},{m}_{2},{u}_{2}\right)}=\left(\frac{{l}_{1}}{{l}_{2}},\frac{{m}_{1}}{{m}_{2}},\frac{{u}_{1}}{{u}_{2}}\right)$$

#### Degree of possibility matrix

The degree of possibility matrix plays a vital role by enabling the comparison of fuzzy numbers to establish the comparative importance of alternatives or criteria. This matrix effectively captures the ambiguity and imprecision inherent in human decision-making by calculating the extent to which one fuzzy number exceeds another, resulting in more precise and dependable decision outcomes. The significance of this matrix has been demonstrated in various recent studies. For example, Patil et al.^43^ employed the degree of possibility to determine criteria weights in a fuzzy context, thereby enhancing the accuracy of decision-making processes. Additionally, research conducted by Liu and Zhang^44^ highlighted how the degree of possibility addresses uncertainties within the Analytic Hierarchy Process, thus improving the stability of priority evaluations. These examples illustrate the matrix’s crucial role in enhancing the consistency and reliability of decisions across multiple domains. The process of evaluating the degree of possibility matrix is done by using Eq. (4).

4 $$\:V\left({S}_{i}\ge\:{S}_{j}\right)=\:\left\{\begin{array}{c}1,\:\:\:\:\:\:\:\:\:\:\:\:\:\:\:\:\:if\:{m}_{i}\ge\:{m}_{j}\\\:\frac{{l}_{i}-{u}_{i}}{\left({m}_{i}-{u}_{i}\right)+\left({m}_{j}-{l}_{j}\right)},\:\:\\\:0,\:if\:{u}_{i}\:\:<\:{l}_{j}\end{array}\right.if\:{u}_{i}\ge\:{l}_{j}\:and\:{m}_{i}<\:{m}_{j}$$

This approach systematically assesses the relative dominance of one fuzzy synthetic extent over another for all pairs of alternatives. In particular:

- For every pair $\:({S}_{i},{S}_{j})$the formula was applied to compute $\:V\left({S}_{i}\:\ge\:{S}_{j}\right)\:$based on the positions of their lower $\:\left(l\right)$, middle $\:\left(m\right)$ and upper $\:\left(u\right)$ values.

- The calculated $\:V\left({S}_{i}\:\ge\:{S}_{j}\right)$ values were stored in the corresponding row $\:\left(i\right)$ and column $\:\left(j\right)$ of the degree of possibility matrix.

- This same formula was uniformly applied across all $\:n\times\:n$pairs of alternatives of irrigation techniques or soil tillage practices.

#### Aggregate the degree of possibility values

For each alternative $\:{S}_{i}\:$compute its overall possibility degree against all other alternatives. This involves calculating the minimum degree of possibility where $\:{S}_{i}\:$dominates other alternatives (Eq. (5)).

5 $$\:{d}^{{\prime\:}}\left({A}_{i}\right)=\:\underset{j\ne\:i}{\text{min}}V({S}_{i}\ge\:{S}_{j})$$

where,

$\:{d}^{{\prime\:}}\left({A}_{i}\right)$: Aggregated possibility degree for alternative $\:{S}_{i}$ 

$\:V({S}_{i}\ge\:{S}_{j})$: Degree of possibility that $\:{S}_{i}$​ dominates $\:{S}_{j}$.

#### Normalize the aggregated values

To obtain the final weights, the combined values for each option are adjusted so that their sum equals one (Eq. (6)). This standardization process ensures that all weights are properly scaled.

6 $$\:{W}_{i}=\:\frac{{d}^{{\prime\:}}\left({A}_{i}\right)}{{\sum\:}_{i=1}^{n}{d}^{{\prime\:}}\left({A}_{i}\right)}\:$$

## Application of fuzzy extent analysis

### Fuzzy synthetic extent calculations

Tables 5 and 6 represent the fuzzy synthetic extent values for various soil tillage methods in relation to multiple criteria using Eqs. (1–3). The values are presented as TFNs for each criterion, with each TFN consisting of three components.

- L (Lower bound): Minimum possible value.
- M (Middle or modal value): Most likely value.
- U (Upper bound): Maximum possible value.

Various soil tillage techniques are assessed based on multiple factors, including cost-effectiveness, crop production, adaptability to climate changes, water retention, soil impact, resistance to diseases, operational simplicity, and efficiency.

According to Table 5, zero tillage receives scores of ZT_L = 0.152, ZT_M = 0.137, and ZT_U = 0.148 for affordability. The minimum value (0.152) indicates that zero tillage is viewed as reasonably affordable in optimal circumstances. The median score (0.137) shows that its typical affordability is slightly below its peak. The maximum value (0.148) implies a limited range of variability, suggesting that perceptions of zero tillage’s affordability remain relatively consistent. Similarly advanced tillage (AT) gets AT_L = 0.070, AT_M = 0.070, AT_U = 0.070 for max yield, with a fixed fuzzy number rating across all three bounds.

**Table 5:** Fuzzy synthetic extent calculations for soil tillage practices.

Criteria	Affordable	Max Yield	Climate Resilient	Water Less Consumption	Less Soil Disturbed	Disease Resistance	Easy Operation	Optimized Nutrient	Promoting Crop Diversification
ZT_L	0.152	0.163	0.179	0.179	0.179	0.161	0.097	0.152	0.161
ZT_M	0.137	0.148	0.158	0.158	0.158	0.143	0.102	0.137	0.143
ZT_U	0.130	0.130	0.137	0.137	0.137	0.134	0.104	0.130	0.134
NT_L	0.152	0.163	0.179	0.179	0.179	0.161	0.097	0.152	0.161
NT_M	0.137	0.148	0.158	0.158	0.158	0.143	0.102	0.137	0.143
NT_U	0.130	0.130	0.137	0.137	0.137	0.134	0.104	0.130	0.134
RT_L	0.091	0.116	0.128	0.128	0.128	0.097	0.161	0.091	0.097
RT_M	0.098	0.115	0.123	0.123	0.123	0.102	0.143	0.098	0.102
RT_U	0.101	0.117	0.123	0.123	0.123	0.104	0.134	0.101	0.104
SM_L	0.091	0.116	0.128	0.128	0.128	0.097	0.097	0.152	0.097
SM_M	0.098	0.115	0.123	0.123	0.123	0.102	0.102	0.137	0.102
SM_U	0.101	0.117	0.123	0.123	0.123	0.104	0.104	0.130	0.104
Cons. Tillage_L	0.091	0.116	0.128	0.128	0.128	0.097	0.097	0.152	0.161
Cons. Tillage_M	0.098	0.115	0.123	0.123	0.123	0.102	0.102	0.137	0.143
Cons. Tillage_U	0.101	0.117	0.123	0.123	0.123	0.104	0.104	0.130	0.134
CTB_L	0.152	0.116	0.077	0.077	0.077	0.097	0.097	0.091	0.097
CTB_M	0.137	0.115	0.088	0.088	0.088	0.102	0.102	0.098	0.102
CTB_U	0.130	0.117	0.096	0.096	0.096	0.104	0.104	0.101	0.104
AT_L	0.091	0.070	0.077	0.077	0.077	0.097	0.161	0.091	0.097
AT_M	0.098	0.082	0.088	0.088	0.088	0.102	0.143	0.098	0.102
AT_U	0.101	0.091	0.096	0.096	0.096	0.104	0.134	0.101	0.104
DT_L	0.030	0.070	0.077	0.077	0.077	0.097	0.097	0.091	0.097
DT_M	0.091	0.116	0.128	0.128	0.128	0.161	0.161	0.152	0.161
DT_U	0.072	0.091	0.096	0.096	0.096	0.104	0.104	0.101	0.104
CT_L	0.152	0.070	0.026	0.026	0.026	0.097	0.097	0.030	0.032
CT_M	0.137	0.082	0.053	0.053	0.053	0.102	0.102	0.059	0.061
CT_U	0.130	0.091	0.068	0.068	0.068	0.104	0.104	0.072	0.075

Table 6 demonstrates that for maximum yield, drip irrigation receives values of DI_L = 0.086, DI_M = 0.170, and DI_U = 0.200. The minimum value (0.086) implies that drip irrigation can have a notable effect on yield in certain circumstances. The median figure (0.170) suggests that, typically, drip irrigation enhances yield considerably when compared to alternative irrigation methods. The maximum value (0.200) indicates that in ideal conditions, drip irrigation can result in significant yield enhancements. Likewise, Precision Irrigation (PRD) is assigned values of PRD_L = 0.086, PRD_M = 0.094, and PRD_U = 0.100 for climate resilience. The consistently low figures across all three fuzzy boundaries suggest that precision irrigation does not play a substantial role in improving climate resilience.

**Table 6:** Fuzzy synthetic extend calculations for irrigation practices.

Criteria	Affordable	Max Yield	Climate Resilient	Water Less Consumption	Less Soil Disturbed	Disease Resistance	Easy Operation	Optimized Nutrient	Promoting Crop Diversification
SI_L	0.086	0.143	0.097	0.116	0.077	0.103	0.097	0.091	0.120
SI_M	0.094	0.132	0.102	0.115	0.088	0.106	0.102	0.098	0.116
SI_U	0.099	0.129	0.106	0.115	0.093	0.108	0.104	0.103	0.115
DI_L	0.086	0.200	0.226	0.163	0.128	0.172	0.097	0.212	0.120
DI_M	0.094	0.170	0.184	0.148	0.123	0.149	0.102	0.176	0.116
DI_U	0.099	0.143	0.152	0.128	0.120	0.138	0.104	0.147	0.115
MI_L	0.086	0.143	0.097	0.116	0.128	0.103	0.097	0.152	0.120
MI_M	0.094	0.132	0.102	0.115	0.123	0.106	0.102	0.137	0.116
MI_U	0.099	0.129	0.106	0.115	0.120	0.108	0.104	0.132	0.115
Alt. FI_L	0.143	0.086	0.097	0.116	0.128	0.103	0.161	0.091	0.120
Alt. FI_M	0.132	0.094	0.102	0.115	0.123	0.106	0.143	0.098	0.116
Alt. FI_U	0.127	0.100	0.106	0.115	0.120	0.108	0.134	0.103	0.115
PRD_L	0.086	0.086	0.161	0.116	0.128	0.103	0.097	0.152	0.120
PRD_M	0.094	0.094	0.143	0.115	0.123	0.106	0.102	0.137	0.116
PRD_U	0.099	0.100	0.136	0.115	0.120	0.108	0.104	0.132	0.115
Def. Irr_L	0.143	0.086	0.097	0.116	0.128	0.103	0.097	0.091	0.120
Def. Irr_M	0.132	0.094	0.102	0.115	0.123	0.106	0.102	0.098	0.116
Def. Irr_U	0.127	0.100	0.106	0.115	0.120	0.108	0.104	0.103	0.115
GPI_L	0.086	0.086	0.097	0.116	0.128	0.103	0.161	0.091	0.120
GPI_M	0.094	0.094	0.102	0.115	0.123	0.106	0.143	0.098	0.116
GPI_U	0.099	0.100	0.106	0.115	0.120	0.108	0.134	0.103	0.115
SIM_L	0.143	0.086	0.097	0.116	0.128	0.103	0.097	0.091	0.120
SIM_M	0.132	0.094	0.102	0.115	0.123	0.106	0.102	0.098	0.116
SIM_U	0.127	0.100	0.106	0.115	0.120	0.108	0.104	0.103	0.115
CFI_L	0.143	0.086	0.032	0.023	0.026	0.103	0.097	0.030	0.040
CFI_M	0.132	0.094	0.061	0.049	0.053	0.106	0.102	0.059	0.070
CFI_U	0.127	0.100	0.076	0.064	0.067	0.108	0.104	0.074	0.082

### Degree of possibility matrix calculations

Tables 7 and 8 represent the degree of possibility matrix for various soil tillage and irrigation methods in relation to the affordability criterion. In similar ways, matrices against other criteria are derived. This matrix is utilized in fuzzy extent analysis to evaluate different tillage techniques and assess their relative affordability compared to one another.

The values range from 0 to 1, where:

- 1 indicates that the affordability of the row tillage practice is fully possible compared to the column tillage practice.
- 0.5 indicates that the affordability of the row practice is moderately possible or equal to the column practice.
- 0 indicates that the affordability of the row practice is less possible compared to the column practice.
- ‘-’ (dash) represents a comparison of the same practice, which is not necessary.

In comparison to other agricultural practices, Zero Tillage (ZT) consistently shows a value of 1(Table 7), indicating its superior affordability. This suggests that ZT is always considered more cost-effective than alternative tillage methods. However, when comparing ZT to Deep Tillage (DT), the value is 0, signifying that ZT is not less affordable than DT.

**Table 7:** Degree of possibility matrix for soil tillage practices against affordability.

Comparison	Zero Tillage (ZT)	No-Tillage (NT)	Reduced Tillage (RT)	Strip Mulching (SM)	Conservation Tillage	Conventional Tillage with Broadcasting (CTB)	Advanced Tillage (AT)	Deep Tillage (DT)	Conventional Tillage (CT)
Zero Tillage (ZT)	-	1	1	1	1	1	1	1	1
No-Tillage (NT)	1	-	1	1	1	1	1	1	1
Reduced Tillage (RT)	0.5	0.5	-	0.5	0.5	0.5	0.5	0.5	0.5
Strip Mulching (SM)	0.5	0.5	0.5	-	0.5	0.5	0.5	0.5	0.5
Conservation Tillage	0.5	0.5	0.5	0.5	-	0.5	0.5	0.5	0.5
Conventional Tillage with Broadcasting (CTB)	1	1	1	1	1	-	1	1	1
Advanced Tillage (AT)	0.5	0.5	0.5	0.5	0.5	0.5	-	0.5	0.5
Deep Tillage (DT)	0	0	0	0	0	0	0	-	0
Conventional Tillage (CT)	1	1	1	1	1	1	1	1	-

According to Table 8, Sprinkler Irrigation (SI) receives a score of 1 when compared to other practices, indicating that it is considered at least as cost-effective as these methods. However, for Surface Irrigation with Mulch (SIM), Deficit Irrigation (Def. Irr.), and Alternate Furrow Irrigation (Alt. FI), Table 8 represents a score of 0. This suggests that both Alt. FI and SIM are viewed as more economical options than SI.

**Table 8:** Degree of possibility matrix for irrigation practices against affordability.

Alternative	Sprinkler Irrigation (SI)	Drip Irrigation (DI)	Micro-sprinkler Irrigation (MI)	Alternate Furrow Irrigation (Alt. FI)	Partial Root-Zone Drying (PRD)	Deficit Irrigation (Def. Irr.)	Gated Pipe Irrigation (GPI)	Surface Irrigation with Mulch (SIM)	Conventional Flood Irrigation (CFI)
Sprinkler Irrigation (SI)	-	1	1	0	1	0	1	0	1
Drip Irrigation (DI)	1	-	1	0	1	0	1	0	1
Micro-sprinkler Irrigation (MI)	1	1	-	0	1	0	1	0	1
Alternate Furrow Irrigation (Alt. FI)	1	1	1	-	1	1	1	1	1
Partial Root-Zone Drying (PRD)	1	1	1	0	-	0	1	0	1
Deficit Irrigation (Def. Irr.)	1	1	1	1	1	-	1	1	1
Gated Pipe Irrigation (GPI)	1	1	1	0	1	0	-	0	1
Surface Irrigation with Mulch (SIM)	1	1	1	1	1	1	1	-	1
Conventional Flood Irrigation (CFI)	1	1	1	0	1	0	1	0	-

## Results and discussion

### Identifying the best irrigation practices

#### Affordability

Research findings suggest that Alternative Furrow Irrigation (Alt. FI), Surface Irrigation with Mulch (SIM), and Deficit Irrigation (Def. Irr.) are the most economical choices (Table 9). According to Kassaye et al.^45^, Alt. FI can decrease water consumption by 30–50% compared to traditional methods without significantly affecting yields, thereby reducing water and energy expenses. Moreover, employing AFI with a 25% reduction in irrigation has resulted in the greatest economic benefits for potato cultivation. Champaneri D et al.^21^ emphasize SIM’s capability to lower evaporation by 20–40% and enhance soil moisture retention, which reduces the need for frequent irrigation. The financial effects of DI in corn farming were assessed, with Donnell et al.^46^ demonstrating that DI, when managed with soil moisture sensors, can lead to substantial water savings while having minimal impact on crop yields, thus benefiting farmers financially. Gated Pipe Irrigation (GPI), Partial Root-Zone Drying (PRD), and Conventional Flood Irrigation (CFI) are also noted for their cost-effectiveness. Iqbal^47^ discovered that PRD conserves 30–50% of water without yield reduction, making it suitable for regions with limited water resources, and GPI enhances water distribution and crop yields. Mebrahtu et al.^48^ and Ebrahimian et al.^49^ indicate that AFI can reduce water usage by 50% compared to Conventional Furrow Irrigation (CFI), while maintaining comparable crop yields and improving Water Use Efficiency (WUE), making it a more cost-effective method.

**Fig. 4:**
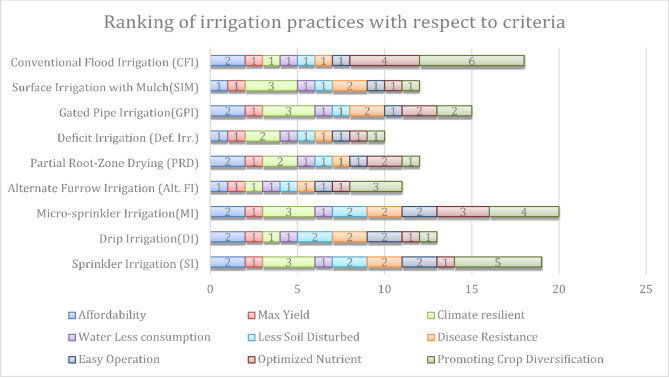
Ranking of irrigation practices with respect to individual criteria.

Alternative Furrow Irrigation (Alt.FI), Deficit Irrigation (DI), and Surface Irrigation with Mulch (SIM) are highly regarded for their cost-effectiveness, primarily due to their low infrastructure needs, decreased energy usage, and minimal maintenance expenses. These methods do away with the necessity for intricate systems like pumps, pipelines, or pressurized setups, thereby greatly reducing both initial investment and ongoing costs. For example, Alt. Furrow Irrigation (Alt.FI) functions by watering every other furrow, naturally halving water and energy consumption. Deficit Irrigation (DI) intentionally restricts water during less critical growth phases of crops, while SIM minimizes evaporation and runoff, resulting in fewer irrigation cycles. Figure 4 illustrates the ranking of individual irrigation practices. The graph shows the effectiveness of these irrigation methods, with smaller bars indicating more efficient techniques.

The results suggest that cost-effective irrigation techniques like Alternative Furrow Irrigation (Alt.FI), Deficit Irrigation (DI), and Surface Irrigation with Mulch (SIM) could be more widely adopted by small-scale and marginal farmers, particularly in arid and semi-arid regions. Policymakers might promote these methods by providing targeted subsidies for mulch materials or by organizing training sessions focused on deficit irrigation planning. Furthermore, integrating these strategies into public irrigation initiatives could enhance economic sustainability at the community level.

#### Max yield

Research findings indicate that various irrigation techniques can yield comparable maximum outputs. All nine irrigation methods assessed showed similar results in terms of crop yield (Table 9). Despite having different ways of delivering water, each system successfully maintained adequate soil moisture during the critical growth phases of the crops, ensuring consistent productivity. Pressurized systems such as DI, SI, and MI deliver water uniformly and precisely near the root zone, which boosts nutrient absorption and minimizes leaching losses. Techniques like PRD and Def. Irr. induce mild and controlled water stress, encouraging deeper root growth and enhancing water-use efficiency. SIM and Alt. FI help reduce evaporation and extend moisture retention through mulching and alternating the wetting of furrows, respectively. Even GPI and CFI, when scheduled optimally, provided enough water to maintain yield levels.

Rao et al.^50^ showed that using micro-sprinkler systems in cultivation led to increases in seedling weight by 19%, height by 16%, root volume by 31%, and the number of transplantable seedlings by 18% when compared to rose-can watering. Li et al.^51^ emphasized the importance of drip and alternate furrow irrigation in enhancing crop yields and water-use efficiency, noting that moderate water deficits can conserve resources without reducing yields. Al-Omran et al.^52^ discovered that combining Partial Root-Zone Drying (PRD) with subsurface drip irrigation enhanced water-use efficiency in potato cultivation. Kassaye et al.^23^ verified that certain levels of deficit irrigation can maintain wheat yield while improving water-use efficiency. Srinivas^20^ highlighted that traditional methods like flood irrigation help stabilize yields and increase agricultural income. Contemporary methods such as drip irrigation and automated systems greatly decrease water usage while either sustaining or improving crop production^53^. Numerous studies have demonstrated that micro-sprinkler irrigation (MI) can greatly enhance both crop yields and water use efficiency. According to research, MI has the potential to boost wheat yields by 5.8–21% when compared to traditional irrigation techniques^54^. The enhanced efficiency in water distribution and application through GPI leads to these advantages. Furrow lengths ranging from 60 to 125 m were identified as ideal for maximizing both yield and water conservation^55^. Recent research has explored how various furrow irrigation methods impact crop yields and water use efficiency. Among these, Conventional Furrow Irrigation (CFI) consistently achieved the highest yields across different crops, such as cabbage, maize and garlic^56,57^.

#### Climate resilient

Out of the nine irrigation methods assessed, Drip Irrigation (DI), Alternate Furrow Irrigation (Alt. FI), and Conventional Flood Irrigation (CFI) emerged as the most resilient to climate variations (Table 9). These systems each bolster a crop’s capacity to endure climatic challenges, albeit through distinct approaches. DI applies precise, low-volume water directly to the root area, ensuring a constant supply of moisture and minimizing the risk of water stress during heatwaves or dry periods. Alt. FI manages soil moisture by alternating between wet and dry furrows, training crops to use water more efficiently while maintaining sufficient hydration. Interestingly, CFI, when executed with proper timing and aligned with crop water needs, sustains high subsoil moisture levels, providing a safeguard against short-term droughts or erratic rainfall, especially for deep-rooted crops like wheat and sugarcane.

Expanding on earlier research, Kumar & Pant^16^ illustrated that both surface and sub-surface DI improve water usage and nutrient distribution, resulting in enhanced crop yields and greater climate resilience. Alternate furrow irrigation (AFI) has become a robust strategy for adapting to climate change in irrigated farming. Research indicates that AFI can cut water usage by 38–50% compared to traditional techniques, while still achieving similar crop yields^16,58,59^. Sarker et al.^60^ identified that Alt. FI increases water productivity without affecting yield, thus enhancing crop resilience. Mwakyusa et al.^61^ highlighted the significance of CFI in boosting soil fertility and crop resilience, despite its potentially lower water-use efficiency. Additionally, Sarker et al.^60^ demonstrated that deficit irrigation enhances water efficiency and improves the quality of tomato fruits, making them more adaptable to climate variations. Iqbal et al.^62^ discovered that Partial Root-Zone Drying (PRD), especially when paired with mulching, maximizes water use and supports sustainable cotton farming in arid regions. Furthermore, techniques like Surface Irrigation with Mulch (SIM), Gated Pipe Irrigation (GPI), and Micro-sprinkler Irrigation (MI) also enhance water use efficiency and resilience to climate change.

The climate resilience provided by DI, Alt. FI, and CFI indicates a varied approach to irrigation planning in response to evolving climate conditions. Alt. FI is recommended for use in row crops and semi-arid regions where both water conservation and adaptability are essential. Collectively, these methods offer a range of resilient irrigation options that can be customized to fit specific local agro-ecological zones.

#### Water less consumption

All nine irrigation methods assessed demonstrated an equal ability to lower water usage when paired with suitable field-level management techniques (Table 9). Pressurized systems such as DI, SI, and MI naturally decrease water consumption by applying it directly where needed, while conservation-oriented systems like SIM, Alt. FI, and PRD reduce losses from evaporation and deep percolation. Moreover, Def. Irr. deliberately restricts irrigation amounts according to the crop’s sensitivity phases. Even methods traditionally known for high water usage, like CFI and GPI, can greatly enhance water-use efficiency when combined with laser land leveling, bunding, precise cut-off timing, and alternate wetting-drying strategies. Therefore, the equal ranking of these practices highlights their adaptability and potential for improvement when applied with care.

Consistent with earlier studies, Drip irrigation is a water-efficient technology that supplies water directly to the roots of plants, minimizing waste and enhancing crop production^63^. Chauhdary et al.^64^ underscored the development and potential of sprinkler and precision irrigation, highlighting their contribution to enhancing water efficiency and crop yields in Asia. Recent advancements in sprinkler systems, including low-pressure sprinklers, intelligent controllers, fertigation, and variable-rate irrigation, present additional possibilities for optimizing water usage and improving application efficiency^65^. Yang et al.^66^ found that adopting drip irrigation can result in crop yield improvements between 3.6% and 66.4%, while also increasing water use efficiency (WUE) by 5.9–60.0%. Iqbal et al.^62^ noted that the combination of partial root-zone drying (PRD) with mulching led to higher cotton yields and water conservation. Li et al.^67^ pointed out the potential of PRD in dry areas and the benefits of surface irrigation with mulch in boosting WUE and yields. Although less efficient, Singh et al.^68^ proposed that controlled flooding and deficit irrigation could enhance WUE with minimal yield losses, depending on the crop type and environmental conditions. According to Mohamed et al.^69^, GPI systems can achieve water application efficiency rates as high as 85.7% in certain instances. The potential to save water is especially crucial for crops that require a lot of water, such as sugarcane. In Egypt, GPI can help conserve more than 1 million cubic meters of water per area^70^.

The consistent performance observed across Sprinkler Irrigation (SI), Drip Irrigation (DI), Micro-sprinkler Irrigation (MI), Alternate Furrow Irrigation (Alt. FI), Partial Root-Zone Drying (PRD), Deficit Irrigation (Def. Irr.), Gated Pipe Irrigation (GPI), Surface Irrigation with Mulch (SIM), and Conventional Flood Irrigation (CFI indicates that various irrigation systems can achieve water conservation objectives when tailored to the specific agro-ecological and infrastructural conditions of a farm. This highlights the necessity of capacity-building, site-specific advisory systems, and policy support for both advanced and basic irrigation techniques. Rather than advocating for a single type of technology, government initiatives and extension programs should empower farmers to select from a diverse range of options, considering factors such as water availability, cost, soil type, and energy access. Encouraging adaptive irrigation strategies at different scales will be crucial for maintaining sustainable agriculture in the face of growing water scarcity.

### Identifying best soil tillage practices

#### Affordability

Among the tillage methods assessed, Zero Tillage (ZT), No-Tillage (NT), Conventional Tillage with Broadcasting (CTB), and Conventional Tillage (CT) stood out as the most cost-effective choices for farmers (Table 10). These approaches demand less investment in machinery and labor compared to more intensive or mechanized options like raised beds, strip tillage, or mechanized precision planting. ZT and NT eliminate the necessity for repeated tillage operations, which in turn lowers expenses related to fuel, labor, and machinery wear and tear. Although Conventional Tillage with Broadcasting (CTB) and Conventional Tillage (CT) involve plowing, they benefit from the widespread familiarity with equipment and existing infrastructure among most smallholder and semi-mechanized farmers, thus reducing the need for additional capital expenditure. According to earlier research, in northwest India, the adoption of ZT wheat farming led to a reduction in production expenses and an increase in net income when compared to traditional tillage methods^71^. In Morocco, fields of ZT wheat produced 23% more and earned 27% higher income than fields that were tilled using conventional methods^72^. Research conducted in Haryana, India, as well as in Nepal and Bihar, India, consistently found that zero tillage (ZT) resulted in higher net income and better benefit-cost ratios compared to conventional tillage (CT). The adoption of ZT led to reduced production expenses by cutting down on labor, machinery, and seed costs^73–75^.

Subsequent to these are Reduced Tillage (RT), Strip Mulching (SM), and Conservation Tillage. Recent studies, including those by Aggarwal et al.^76^ and Liang et al.^77^, suggest that conservation tillage, particularly Zero Tillage (ZT) and No-Till (NT), boosts resource use efficiency and economic returns when combined with crop diversification in maize-soybean systems. Singh et al.^78^ found that conservation tillage enhances soil water retention and reduces erosion, leading to higher yields and improved economic efficiency in dryland areas. Deleon et al. highlighted that conservation tillage increases soil biodiversity, which subsequently enhances soil health, water dynamics, and ultimately farm incomes in furrow-irrigated systems.

The cost-effectiveness of Zero Tillage (ZT), No-Tillage (NT), Conventional Tillage with Broadcasting (CTB), and Conventional Tillage (CT) make these methods highly accessible and practical for a broad spectrum of farming communities, particularly for small and marginal farmers. These techniques help cut down expenses related to fuel, labor, machinery use, and repeated field preparations, ensuring economic sustainability both in the short and long term. For stakeholders like agricultural extension agencies, cooperatives, and rural development organizations, advocating for these affordable tillage systems offers a scalable means to support farming livelihoods without imposing significant financial burdens. In essence, these tillage methods provide a balanced strategy—delivering immediate economic benefits to farmers while also establishing a foundation for more resource-efficient and sustainable land management practices.

**Fig. 5:**
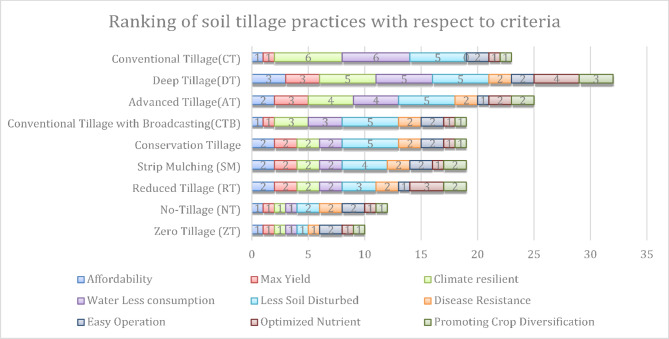
Ranking of soil tillage practices with respect to individual criteria.

#### Max yield

In terms of maximizing crop yields, various tillage techniques have shown to be effective. Among these, Zero Tillage (ZT), No-Tillage (NT), Conventional Tillage with Broadcasting (CTB), and Conventional Tillage (CT) are recognized as the most productive approaches (Table 10). Recent research supports the effectiveness of Zero tillage (ZT) or no-tillage (NT) methods in enhancing crop yields and soil quality when compared to traditional tillage (CT). Meta-analyses and reviews reveal that ZT/NT notably boosts soil organic carbon levels, particularly in the uppermost 10 cm of soil^79,80^. According to Hussain et al.^81^, in a mung bean-wheat cropping system in Punjab, Pakistan, zero tillage led to wheat and mung bean yields that were 13% and 9% higher, respectively, compared to conventional tillage.

A meta-analysis revealed that strip tillage, a type of CT, resulted in a 4.81% increase in crop yields compared to no-tillage^82^. In China, CT led to a yield increase of at least 4–6% across various cropping systems^83^. On a global scale, adopting CT for more than three years resulted in an average crop yield increase of 3.7%, with notable enhancements observed in North America and Australia/Oceania^84^. Chauke et al.^85^ found that no-till methods significantly increased soybean yields due to improved nutrient cycling, enhanced water retention, and reduced soil erosion, with the benefits being particularly noticeable during drier periods. Reduced Tillage (RT), Strip Mulching (SM), and Conservation Tillage also showed high productivity. Over two growing seasons, conventional tillage (T1) resulted in greater plant height, cob weight, and 1000-grain weight compared to reduced (T2) and no-tillage (T3) treatments. Similarly, Song et al.^86^ observed that traditional tillage with straw incorporation notably boosted rice yields. A meta-analysis by Huang et al.^4^ indicated that crop yields increased with tillage depths ranging from 25 to 35 cm, peaking at 35 cm before declining, suggesting an optimal depth for maximizing yield. In sub-Saharan Africa, reduced tillage significantly improved yields by enhancing soil fertility, water retention, and crop resilience, particularly in areas with poor soil and erratic rainfall. Research by Huang et al.^87^ showed that using straw strip mulching techniques led to a 6.90% increase in winter wheat production compared to conventional planting methods, attributed to better soil moisture retention and more efficient water use throughout the growth cycle.

Advanced Tillage (AT) and Deep Tillage (DT) are ranked third in terms of maximizing yields. According to Sadiq et al.^88^, advanced tillage improved the physical, chemical, and biological properties of soil in spring wheat agroecosystems, leading to better yields and a reduction in greenhouse gas emissions. Huang et al.^4^ found that deep tillage, when performed at optimal depths, significantly increased crop production, although this effect decreased at greater depths.

The impressive yield results of Zero Tillage (ZT), No-Tillage (NT), Conventional Tillage with Broadcasting (CTB), and Conventional Tillage (CT) underscore their significance in enhancing agricultural productivity across diverse farming systems. These methods provide farmers with reliable options to attain high crop yields while accommodating their specific resources, soil conditions, and cropping patterns. Their versatility and efficiency make them particularly pertinent in both established and evolving agricultural areas.

#### Climate resilient

Among climate-resilient agricultural practices, Zero Tillage (ZT) and No-Tillage (NT) are considered the most effective (Table 10). Zero Tillage (ZT) and No-Tillage (NT) are regarded as climate-resilient practices because they preserve and enhance the soil’s natural structure and biological activity, which are essential for protecting crops from extreme weather conditions. By reducing soil disturbance, these methods encourage greater retention of organic matter, improved water infiltration, and moisture conservation, making the soil more resilient to droughts and erratic rainfall. Furthermore, they help decrease erosion and maintain surface residues, which serve as protective layers against heat stress and excessive evaporation. Over time, ZT and NT foster a more stable and resilient agroecosystem, allowing crops to perform more reliably even amidst changing climate conditions.

Building on earlier research, Zero tillage (ZT) has been recognized as a climate-resilient farming method that offers numerous advantages. ZT enhances soil structure, increases organic matter, and boosts water use efficiency, while also minimizing erosion and greenhouse gas emissions^80^. No-till farming systems exhibit enhanced drought resilience, as they sustain higher levels of soil moisture and ensure stable grain yields even under water scarcity^89^. Integrating cover crops such as rye with NT enhances soil organic carbon and improves water retention, making it a valuable approach for reducing the effects of climate change on agriculture^79^. Devi et al. illustrated that adopting no-tillage techniques, along with maintaining surface residue and utilizing cover crops, significantly improved soil health in the San Joaquin Valley over a long duration. The researchers concluded that conservation tillage is an effective strategy to boost climate resilience in agricultural systems. Additionally, Dong et al.^5^ found that extended zero-tillage practices enhance soil carbon retention, thereby improving soil health and resilience. In terms of climate resilience, Reduced Tillage (RT), Strip Mulching (SM), and Conservation Tillage are prioritized in descending order of significance (Table 10). Conventional Tillage with Broadcasting (CTB) ranks third in climate resilience, followed by Advanced Tillage (AT) and Deep Tillage (DT) in fourth and fifth places, respectively. Conventional Tillage (CT) is deemed the least effective method for cultivating climate-resilient crops. According to Steponavičienė et al.^90^, conventional tillage (CT) is less effective in promoting climate-resilient crops compared to conservation tillage methods. Their research indicated that CT can lead to soil degradation and reduced crop yields over time, thus not supporting the growth of climate-resilient crops.

The climate-resilient characteristics of Zero Tillage (ZT), No-Tillage (NT), are particularly beneficial for farmers dealing with increasingly erratic weather conditions. These methods not only enhance crop survival during periods of drought or excessive rainfall but also promote long-term sustainability by preserving soil moisture and minimizing degradation. For agricultural planners and development organizations, advocating for Zero Tillage (ZT), No-Tillage (NT), is a strategic move towards climate-smart agriculture. Training initiatives, field demonstrations, and policy backing centered on these tillage techniques can aid farming communities in building resilience to climate disruptions while enhancing soil health and productivity over time. Their implementation can also significantly contribute to national goals related to climate adaptation, land restoration, and sustainable agriculture.

#### Water less consumption

Studies show that Zero Tillage (ZT) and No-Tillage (NT) are considered some of the most effective tillage methods for conserving water (Table 10). Zero Tillage (ZT) and No-Tillage (NT) are regarded as highly efficient methods for conserving water because they uphold soil integrity, conserve crop residues, and minimize evaporation. By reducing or eliminating soil disruption, these techniques aid in prolonging soil moisture retention, decreasing runoff, and enhancing water infiltration. The layer of residue on the soil surface serves as a protective barrier, shielding the soil from direct sunlight and lessening the need for frequent irrigation. Consequently, Zero Tillage (ZT) and No-Tillage (NT) are particularly suitable for regions experiencing water shortages or unpredictable rainfall, providing a viable approach to boost water-use efficiency in agriculture.

For instance, Zero tillage (ZT) and no-tillage (NT) techniques have demonstrated their ability to conserve water in dryland farming systems. NT improves soil moisture retention, boosts grain production, and enhances water use efficiency in wheat and maize cultivation across a range of soil types and weather conditions^91^. Zero tillage (NT) has demonstrated its ability to conserve water and enhance water productivity across different agricultural systems. It minimizes evaporation, boosts water use efficiency, and sustainably increases crop yields^92^. Haruna et al.^79^ illustrated that incorporating cover crops into no-till systems improved soil water infiltration, thereby enhancing water conservation. Furthermore, Sairam et al.^93^ found that conservation tillage methods, such as zero and reduced tillage, improve soil health by boosting its physical, chemical, and biological properties, leading to better water retention and reduced erosion. These findings align with previous studies, showing that ZT and NT methods effectively maintain soil moisture, thus supporting sustainable farming practices. (Table 10). Among these methods, Conventional Tillage (CT) is considered the least efficient in terms of water use. Research by Fatumah et al.^94^ supported this by demonstrating that no-tillage and stubble-mulching techniques significantly enhance soil water retention and increase common bean yields compared to traditional tillage methods. These techniques also improved water use efficiency and grain yields. Notably, the no-tillage approach surpassed other methods, producing yields that were over 5%, 38%, and 43% higher than those achieved with stubble-mulching, deep tillage, and conventional tillage, respectively.

Zero Tillage (ZT) and No-Tillage (NT) capacity to conserve water is especially crucial for farmers working in areas with limited water resources. These methods present a cost-effective and sustainable alternative to farming that relies heavily on irrigation, thereby alleviating the strain on groundwater and canal systems. For stakeholders like policymakers, NGOs, and extension services, advocating for water-efficient tillage practices such as ZT and NT can aid in achieving broader objectives like water conservation, drought readiness, and sustainable intensification. Their implementation can be expedited through demonstration plots, awareness initiatives, and incorporation into watershed management and climate adaptation strategies. Ultimately, these practices bolster both environmental stewardship and enhance farm resilience amid increasing water-related challenges.

### Overall ranking of the parameters

#### Soil tillage practices

According to the findings, Zero Tillage (ZT) emerges as the leading agricultural method when assessed on various factors such as cost-effectiveness, maximizing crop yield, climate adaptability, water conservation, minimal soil disturbance, disease resistance, ease of operation, efficient nutrient use, and enhancement of crop diversity (Table 9).

It is deemed highly cost-effective as it removes the necessity for repeated plowing, harrowing, and land preparation tasks, thereby significantly cutting down on expenses related to fuel, labor, and machinery. Farmers can plant directly into the remnants of the previous crop using a ZT drill, bypassing expensive field operations and conserving valuable time during the sowing period. Beyond its financial advantages, ZT enhances soil structure, boosts water retention, minimizes erosion, and fosters microbial activity. These combined effects render ZT both an economically feasible and environmentally sustainable option for farmers across diverse resource levels and agro-ecological settings.

Zero Tillage (ZT) is recognized as a prominent technique for sustainable agriculture, as supported by earlier research. It contributes to better soil health, enhances soil structure, and boosts the content of organic matter^15,80^. By enhancing water use efficiency and carbon sequestration, it boosts crop yields, strengthens food security, and increases environmental resilience^35^. ZT adoption is further encouraged by climate change factors, as it contributes to both climate mitigation and adaptation^95^. Hussain et al.^57^ showed that zero tillage (ZT) greatly boosts crop yields and economic gains in mung bean-wheat cropping systems, presenting a cost-effective alternative to conventional tillage methods. According to Sadiq et al.^88^, no-till farming enhances the soil’s physical, chemical, and biological properties, leading to improved water retention and greater resilience to climate changes, which are vital for sustainable agriculture. Liang et al.^77^ found that ZT reduces soil disturbance, enhances soil structure, and lowers the occurrence of soil-borne diseases, thereby supporting long-term soil health and sustainable productivity. Additionally, Haruna et al.^96^ noted that no-tillage improves nutrient cycling and streamlines farm operations while maintaining high productivity levels. Collectively, these studies affirm that ZT is a superior method for sustainable farming, providing environmental, economic, and agronomic advantages that make it an ideal practice for contemporary agriculture^88^. Research also indicates that Deep Tillage (DT) is less effective in all aspects. DT generally leads to higher operational costs due to increased fuel use and labor demands. While it may temporarily alleviate soil compaction, DT disrupts soil structure, causing more surface runoff and soil erosion, which ultimately diminishes long-term soil fertility and crop yields.

Zero Tillage (ZT) is a fundamental practice in promoting sustainable agriculture due to its extensive benefits. It offers farmers a cost-effective way to sustain high yields while lowering expenses and reducing environmental harm. Zero Tillage (ZT) enhances the efficient use of water, energy, and fertilizers, and boosts soil health, making it particularly beneficial in areas with limited resources and climate challenges. For those involved in agriculture and policy-making, advocating for Zero Tillage (ZT) is a practical strategy to tackle food security, environmental protection, and climate resilience. Expanding its use can be facilitated through focused training, peer-to-peer farmer outreach, shared equipment models, and local demonstrations. With its wide-ranging benefits, Zero Tillage (ZT) has significant potential to enhance both agricultural productivity and ecological sustainability on a broad scale.

**Table 9:** Overall ranking of soil tillage practices.

Alternative	Affordability	Max Yield	Climate resilient	Water Less consumption	Less Soil Disturbed	Disease Resistance	Easy Operation	Optimised Nutrients	Promoting Crop Diversification	Final Aggregated Score	Rank
Zero Tillage (ZT)	0.030	0.029	0.022	0.026	0.020	0.025	0.009	0.006	0.009	0.176	1
No-Tillage (NT)	0.030	0.029	0.022	0.026	0.016	0.025	0.009	0.006	0.009	0.172	2
Reduced Tillage (RT)	0.015	0.015	0.017	0.020	0.009	0.013	0.010	0.003	0.005	0.105	4
Strip Mulching (SM)	0.015	0.015	0.017	0.020	0.006	0.013	0.009	0.006	0.005	0.104	5
Conservation Tillage	0.015	0.015	0.017	0.020	0.000	0.013	0.009	0.006	0.009	0.101	6
Conventional Tillage with Broadcasting (CTB)	0.030	0.029	0.008	0.010	0.000	0.013	0.009	0.006	0.009	0.113	3
Advanced Tillage (AT)	0.015	0.000	0.006	0.007	0.000	0.013	0.010	0.003	0.005	0.058	8
Deep Tillage (DT)	0.000	0.000	0.003	0.003	0.000	0.013	0.009	0.000	0.000	0.027	9
Conventional Tillage (CT)	0.030	0.029	0.000	0.000	0.000	0.013	0.009	0.006	0.009	0.095	7

**Table 10:** Overall ranking of irrigation practices.

Alternative	Affordability	Max Yield	Climate resilient	Water Less consumption	Less Soil Disturbed	Disease Resistance	Easy Operation	Optimized Nutrient	Promoting Crop Diversification	Final Aggregated Score	Rank
Sprinkler Irrigation (SI)	0.017	0.018	0.007	0.014	0.004	0.011	0.006	0.007	0.009	0.092	8
Drip Irrigation (DI)	0.017	0.018	0.018	0.014	0.004	0.011	0.006	0.007	0.016	0.111	6
Micro-sprinkler Irrigation (MI)	0.017	0.018	0.007	0.014	0.004	0.011	0.006	0.001	0.009	0.086	9
Alternate Furrow Irrigation (Alt. FI)	0.027	0.018	0.018	0.014	0.015	0.022	0.012	0.007	0.010	0.143	2
Partial Root-Zone Drying (PRD)	0.017	0.018	0.014	0.014	0.015	0.022	0.012	0.003	0.016	0.130	3
Deficit Irrigation (Def. Irr.)	0.027	0.018	0.014	0.014	0.015	0.022	0.012	0.007	0.016	0.144	1
Gated Pipe Irrigation (GPI)	0.017	0.018	0.007	0.014	0.015	0.011	0.006	0.003	0.011	0.101	7
Surface Irrigation with Mulch (SIM)	0.027	0.018	0.007	0.014	0.015	0.011	0.006	0.007	0.016	0.120	4
Conventional Flood Irrigation (CFI)	0.017	0.018	0.018	0.014	0.015	0.022	0.012	0.000	0.000	0.116	5

#### Irrigation practices

Deficit Irrigation (DI) emerged as the most effective irrigation method when evaluated against all major sustainability criteria, such as yield, water usage, cost-effectiveness, climate adaptability, energy conservation, and reduced reliance on inputs like fertilizers and pesticides (Table 10). Deficit Irrigation (DI) is distinguished by its deliberate restriction of water application during less critical phases of crop development, enabling plants to adjust while optimizing water efficiency. This method not only saves water but also cuts down on energy needed for pumping, minimizes the risk of nutrient runoff, and can even induce stress-related physiological changes that improve crop quality. From a cost perspective, Deficit Irrigation (DI) can be adopted using current infrastructure with only minor adjustments to irrigation timing, making it financially viable for a broad spectrum of farmers.

Building on prior research, Deficit Irrigation (DI) has been recognized as an effective water management approach in agriculture, delivering advantages for a range of crops and environments. DI entails supplying less water than the crop’s full needs, with the goal of enhancing water productivity^97,98^. In the case of vegetables, DI can improve water efficiency and the quality of produce after harvest, with only slight reductions in yield^21^. Hassan et al.^80^ illustrated that deficit irrigation (DI) methods can greatly improve water productivity in a range of crops. Their research indicated that DI sustains high water productivity while achieving satisfactory yields, effectively balancing water conservation with crop production in pomegranate cultivation. Sarker et al.^60^ emphasized the advantages of DI in rice farming, highlighting notable water savings without yield reduction. Xu et al.^99^ found that DI can enhance water use efficiency (WUE) by 7.39% while causing a 15% decrease in yield, suggesting a beneficial trade-off between water conservation and crop production. The study further indicates that using optimal management strategies, such as suitable irrigation intervals and nitrogen applications, can minimize yield losses and increase economic returns, demonstrating the cost-effectiveness of DI. Moreover, the research highlights DI’s contribution to enhancing crop resilience in dry regions, improving soil moisture retention, and accommodating different cotton varieties, thereby promoting sustainable agricultural practices.

Alternate Furrow Irrigation (Alt. FI) is the second most effective method, with Partial Root-Zone Drying (PRD) coming in third. As supported by earlier research, AFI consistently shows greater water use efficiency than other irrigation methods for a range of crops, including garlic, cabbage, and maize^56,57^. Surface Irrigation with Mulch (SIM) holds the fourth spot, while Conventional Flood Irrigation (CFI) is in fifth place. Drip Irrigation (DI) ranks sixth, followed by Gated Pipe Irrigation (GPI) in seventh. Sprinkler Irrigation (SI) and Micro-sprinkler Irrigation (MI) are in eighth and ninth positions, respectively. Figure 5 illustrates the ranking of individual soil tillage practices. The graph shows the effectiveness of these tillage methods, with smaller bars indicating more efficient techniques.

Deficit Irrigation (DI) excels in agronomic, environmental, and economic aspects, making it a highly scalable and effective strategy for sustainable water management in agriculture. For farmers, DI provides a viable way to conserve water, lower energy expenses, and sustain crop yields, even when faced with climate-related water shortages. Its compatibility with both modern and traditional irrigation systems facilitates its implementation across various farm sizes and cropping patterns. For stakeholders, advocating for Deficit Irrigation (DI) offers a chance to optimize water distribution, mitigate groundwater depletion, and enhance resilience in regions with limited water resources. Investing in farmer education, awareness initiatives, and straightforward monitoring tools can promote the widespread adoption of Deficit Irrigation (DI) as a fundamental practice for water-efficient agriculture.

### Suitable combinations of irrigation and soil tillage practices

The research recommends combining soil management practices with irrigation strategies to meet specific target criteria.

#### Affordability

Research findings indicate that several tillage techniques, including Zero Tillage (ZT), No-Tillage (NT), Conventional Tillage with Broadcasting (CTB), and Conventional Tillage (CT), are the most cost-effective options (Table 11). Previous research supports the notion that combining Zero-tillage (ZT) with deficit irrigation (Def. Irr.) can decrease expenses by reducing labor and water consumption while sustaining consistent wheat yields, thereby offering financial advantages across various growing season climates in the Eastern Indo-Gangetic Plains. Adopting zero-tillage (ZT) and deficit irrigation (DI) methods can improve both agricultural sustainability and economic returns in regions with scarce water resources. ZT enhances soil moisture retention, which can lead to a 23% increase in wheat yield and a 27% rise in profits compared to conventional tillage practices, making this approach cost-effective^72,100^. Nilahyane et al.^100^, discovered that implementing deficit irrigation within a no-till system not only conserved water but also lowered production costs, while simultaneously providing environmental benefits. This approach improves water use efficiency and diminishes the need for labor and fuel typically associated with conventional tillage practices. Nilahyane et al.^100^, also examined Fatumah et al.^94^’s findings, which showed that the combination of No Tillage (NT) and Surface irrigation with Mulch (SIM) resulted in enhanced water use efficiency and grain yield, generating a net profit three times greater than that of conventional tillage.

#### Max yield

Recent studies highlight the effectiveness of different tillage techniques in enhancing crop yields, including Zero Tillage (ZT), No-Tillage (NT), Conventional Tillage with Broadcasting (CTB), and Conventional Tillage (CT) (Table 11). Building on earlier research, Numerous studies have demonstrated that no-tillage (NT) combined with surface mulching offers considerable advantages for both crop yield and soil health. Specifically, NT using leguminous green manure as mulch decreased soil evaporation by 12.8% and boosted maize production by 34.3% in comparison to traditional tillage methods^101^. In cotton farming, using surface mulching with no-till (NT) methods led to a 23.0% increase in seed yield compared to NT without mulching^91^. Fatumah et al.^94^ showed that combining No-Tillage (NT) with Surface Irrigation with Mulch (SIM) leads to better harvests. This method resulted in significantly higher grain production, due to improved soil structure and water retention. Yield consistency also increased, as the mulch covering helped regulate temperature fluctuations, which is especially advantageous in dry and semi-dry regions. In another study, Kumar et al.^40^ explored the combination of Zero Tillage (ZT) and Gated Pipe Irrigation (GPI). Their research revealed that this pairing significantly improved yield-related parameters such as test weight, spike length, and productive tillers. The resulting grain yield was one of the highest observed among various treatments, suggesting that this approach can effectively increase crop productivity.

**Table 11:** Combination of both irrigation and soil tillage practices.

Criteria	Soil Tillage Practices	Irrigation Practices
Affordability	Zero Tillage (ZT), No-Tillage (NT), Conventional Tillage with Broadcasting (CTB), Conventional Tillage (CT)	Alternate Furrow Irrigation (Alt. FI), Deficit Irrigation (Def. Irr.), Surface Irrigation with Mulch (SIM)
Max Yield	Zero Tillage (ZT), No-Tillage (NT), Conventional Tillage with Broadcasting (CTB), Conventional Tillage (CT)	Sprinkler Irrigation (SI), Drip Irrigation (DI), Micro-sprinkler Irrigation (MI), Alternate Furrow Irrigation (Alt. FI), Partial Root-Zone Drying (PRD), Deficit Irrigation (Def. Irr.), Gated Pipe Irrigation (GPI), Surface Irrigation with Mulch (SIM), Conventional Flood Irrigation (CFI),
Climate resilient	Zero Tillage (ZT), No-Tillage (NT),	Drip Irrigation (DI), Alternate Furrow Irrigation (Alt. FI), Conventional Flood Irrigation (CFI)
Water Less consumption	Zero Tillage (ZT), No-Tillage (NT),	Sprinkler Irrigation (SI), Drip Irrigation (DI), Micro-sprinkler Irrigation (MI), Alternate Furrow Irrigation (Alt. FI), Partial Root-Zone Drying (PRD), Deficit Irrigation (Def. Irr.), Gated Pipe Irrigation (GPI), Surface Irrigation with Mulch (SIM), Conventional Flood Irrigation (CFI),
Less Soil Disturbed	Zero Tillage (ZT),	Alternate Furrow Irrigation (Alt. FI), Partial Root-Zone Drying (PRD), Deficit Irrigation (Def. Irr.), Gated Pipe Irrigation (GPI), Surface Irrigation with Mulch (SIM), Conventional Flood Irrigation (CFI),
Disease Resistance	Zero Tillage (ZT), No-Tillage (NT),	Alternate Furrow Irrigation (Alt. FI), Partial Root-Zone Drying (PRD), Deficit Irrigation (Def. Irr.), Conventional Flood Irrigation (CFI)
Easy Operation	Reduced Tillage (RT), Advanced Tillage (AT)	Alternate Furrow Irrigation (Alt. FI), Partial Root-Zone Drying (PRD), Deficit Irrigation (Def. Irr.), Gated Pipe Irrigation (GPI), Surface Irrigation with Mulch (SIM), Conventional Flood Irrigation (CFI),
Optimized Nutrient	Zero Tillage (ZT), No-Tillage (NT), Conventional Tillage with Broadcasting (CTB), Conventional Tillage (CT), Strip Mulching (SM), Conservation Tillage	Sprinkler Irrigation (SI), Drip Irrigation (DI), Alternate Furrow Irrigation (Alt. FI), Deficit Irrigation (Def. Irr.), Surface Irrigation with Mulch (SIM)
Promoting Crop Diversification	Zero Tillage (ZT), No-Tillage (NT), Conventional Tillage with Broadcasting (CTB), Conventional Tillage (CT), Conservation Tillage	Drip Irrigation (DI), Partial Root-Zone Drying (PRD), Deficit Irrigation (Def. Irr.), Surface Irrigation with Mulch (SIM)

#### Climate resilient

Research demonstrates that conservation tillage techniques, such as Zero Tillage (ZT) and No-Tillage (NT), contribute to the development of crops with increased resilience to climate change (Table 11). Kakraliya et al.^102^ confirmed previous findings, showing that the combination of subsurface drip irrigation and no-till practices resulted in higher wheat grain yields compared to traditional tillage with flood irrigation. This integration created optimal conditions for crop growth and development, leading to improved productivity and ultimately more climate-resilient crops. Integrating no-tillage (NT) systems with drip irrigation and fertigation has yielded encouraging outcomes for a variety of crops. Research suggests that NT combined with drip irrigation can notably enhance nitrogen absorption, grain protein levels, and wheat yield when compared to conventional tillage practices, thereby supporting climate resilience in crops^103,104^. Cárceles et al.^105^ explored the effects of conservation agriculture practices, including NT and DI, on soil health. Their research indicated that combining these methods can enhance soil structure, boost organic matter content, and foster sustainable agricultural systems, all of which benefit plant health and promote climate resilience in crops. Although NT might initially have negative impacts on wheat yields in irrigated areas, significant improvements in yield, radiation interception, and biomass production can be achieved by optimizing water and nitrogen management through drip irrigation^104^. Acevedo et al.^106^ examined the impact of soil health practices on water dynamics in irrigated systems. Their study revealed that techniques like No-Tillage (NT) can enhance water availability by improving infiltration and soil water retention. When used in conjunction with efficient irrigation methods such as Drip Irrigation (DI), these practices can optimize productive water flows, minimize evaporation, and support crop growth, thereby enhancing plant health.

#### Water less consumption

Research suggests that conservation tillage practices, such as Zero Tillage (ZT) and No-Tillage (NT), help reduce water consumption in agriculture (Table 11). Fatumah et al.^94^ confirmed previous findings that combining No-Tillage (NT) with Surface Irrigation and Mulch (SIM) effectively enhances water-use efficiency. Research has shown that using NT with deficit drip irrigation can enhance the efficiency of irrigation water usage by 22–48% compared to traditional techniques, while keeping yields consistent and minimizing the risk of nitrate leaching^107^. In wheat-maize cropping systems, the adoption of conservation tillage and improved irrigation techniques like drip or subsurface irrigation led to a significant reduction in water consumption compared to traditional methods, while also boosting soil moisture and organic carbon content^40^. NT maintains soil structure by minimizing disturbance, facilitating improved water infiltration and retention in the root zone. Mulching creates a protective soil cover, decreasing evaporation and boosting moisture availability for crops, with mulched no-tillage fields retaining 30–50% more soil moisture. Nilahyane et al.^100^demonstrated that applying deficit irrigation in sugar beet cultivation decreased water requirements and improved water use efficiency. Integrating Zero Tillage (ZT) with Deficit Irrigation (Def. Irr.) can effectively optimize water usage. Odhafa et al.^108^, assessed tillage systems for wheat production under various irrigation methods and found that Zero Tillage (ZT) with sprinkler irrigation enhanced soil water retention efficiency by 14% compared to conventional tillage. Singh et al.^98^, reported that the combination of Zero Tillage (ZT) and Sprinkler Irrigation (SI) conserved 43.3% of irrigation water relative to traditional tillage without residue retention. Kumar et al.^26^, examined the pairing of Zero Tillage (ZT) with Gated Pipe Irrigation (GPI) and discovered that it reduced soil disruption while utilizing gated pipes for regulated water delivery, resulting in decreased water loss and enhanced water-use efficiency. The controlled water application through gated pipes ensures direct delivery to the root zone, minimizing evaporation and runoff losses.

## Conclusions

This research effectively showcases how combining soil tillage and irrigation techniques can lead to sustainable agricultural practices. By employing a fuzzy synthetic analysis, the study reveals that Zero Tillage (ZT) is the most effective soil tillage method across various factors, including affordability, max yield, climate resilience, water less consumption, less soil disturbance, disease resistance, easy operation, optimized nutrients, and crop diversification. Likewise, Deficit Irrigation emerges as the top irrigation method due to its affordability, water conservation benefits, and potential to boost crop resilience and nutrient uptake. The research emphasizes the need to adopt customized combinations of tillage and irrigation methods to achieve specific farming goals. The integration of data-driven insights, expert input, and fuzzy methodologies provides a robust framework for addressing uncertainties in decision-making processes. However, the study’s reliance on a limited number of base research papers for parameter extraction and qualitative comparison is a notable limitation. This constraint may impact the comprehensiveness and applicability of the results, suggesting that future studies should incorporate a wider range of literature to enhance the analysis’s robustness. The method relies significantly on expert-generated triangular fuzzy numbers, which, although useful, might not adequately represent the nonlinear intricacies of interactions among sustainability parameters. Furthermore, the method of pairwise comparisons presumes that criteria are independent, which could lead to an oversimplification of the interdependencies present in integrated agricultural systems.

The findings ultimately facilitate informed decision-making for farmers and stakeholders, promoting practices that optimize resource use, increase productivity, and ensure environmental sustainability. Future research could explore the implications of these practices in various agro-climatic zones and investigate advanced technologies to further refine the assessment and implementation of sustainable farming methods.

## Supplementary Information

Below is the link to the electronic supplementary material.

Supplementary Material 1

## Data Availability

The datasets analyzed during the current study are not publicly available due to ethical concerns regarding privacy and the protection of personal data but are available from the corresponding author on reasonable request.

## Declarations

### All methods including procuring expert opinions were carried out in accordance with relevant guidelines and regulations

by the Department of Civil Engineering, Birla Institute of Technology and Science, Pilani, India.

### All experimental protocols (procuring expert opinion)

were approved by the Department of Civil Engineering, Birla Institute of Technology and Science, Pilani, India.
