# Supplementary material for: Optimizing water-efficient agriculture: evaluating the sustainability of soil management and irrigation synergies using fuzzy extent analysis
Source: Sci Rep. 2025 Aug 11;15:29382. doi: 10.1038/s41598-025-15426-6 (PMC12339989; doi:10.1038/s41598-025-15426-6)
Supplement: Supplementary file 1 — Supplementary Material 1 [file 41598_2025_15426_MOESM1_ESM.docx]

**Supplementary**

**Optimizing water-efficient agriculture: Evaluating the sustainability of soil management and irrigation synergies using fuzzy extent analysis**

**Rishikesh Sharma^1*^, Srinivas Rallapalli ^1,2*^, Joe Magner^2^**

^1^Department of Civil Engineering, Birla Institute of Technology and Science, Pilani, Pilani Campus, Vidya Vihar, Pilani, Rajasthan 333031, India

^2^Department of Bioproducts and Biosystems Engineering, University of Minnesota, Twin cities, USA

*Corresponding authors emails: [rishikesh.sharma@pilani.bits-pilani.ac.in](mailto:rishikesh.sharma@pilani.bits-pilani.ac.in), [r.srinivas@pilani.bits-pilani.ac.in](mailto:r.srinivas@pilani.bits-pilani.ac.in)

**Section A: Questionnaire**

**Expert Questionnaire for Evaluating Soil Tillage and Irrigation Practices**

**Title**: *Evaluation of Irrigation and Tillage Practices for Sustainable Agriculture Using Fuzzy Extent Analysis***Instructions:**You are requested to rate each irrigation and soil tillage practice against the nine decision-making criteria using the following linguistic scale:

- Very Good (VG)
- Good (G)
- Moderate (M)
- Poor (P)
- Very Poor (VP)

**Please provide your ratings based on your domain knowledge and/or practical experience.**

**Part 1: Irrigation Practices Evaluation**

For each irrigation method listed below, kindly rate its performance with respect to each criterion.

| \| **Survey number________** \|  \| \| --- \| --- \| \| **Name:                                                                                             Designation/Profession:    Academic qualification:**  **Experience:** \| \| | | | | | | | | | |
| --- | --- | --- | --- | --- | --- | --- | --- | --- | --- | --- | --- | --- | --- |
| Criteria \ Irrigation Method | SI | DI | MI | Alt. FI | PRD | Def. Irr. | GPI | SIM | CFI |
| 1. Affordability |  |  |  |  |  |  |  |  |  |
| 2. Maximum Yield |  |  |  |  |  |  |  |  |  |
| 3. Climate Resilience |  |  |  |  |  |  |  |  |  |
| 4. Water Consumption (Less is better) |  |  |  |  |  |  |  |  |  |
| 5. Soil Disturbance (Less is better) |  |  |  |  |  |  |  |  |  |
| 6. Disease Resistance |  |  |  |  |  |  |  |  |  |
| 7. Ease of Operation |  |  |  |  |  |  |  |  |  |
| 8. Nutrient Optimization |  |  |  |  |  |  |  |  |  |
| 9. Crop Diversification Promotion |  |  |  |  |  |  |  |  |  |

**Part 2: Soil Tillage Practices Evaluation**

Please rate each tillage practice against the same set of criteria.

| Criteria \ Tillage Method | ZT | NT | RT | SM | Cons. Tillage | CTB | AT | DT | CT |
| --- | --- | --- | --- | --- | --- | --- | --- | --- | --- |
| 1. Affordability |  |  |  |  |  |  |  |  |  |
| 2. Maximum Yield |  |  |  |  |  |  |  |  |  |
| 3. Climate Resilience |  |  |  |  |  |  |  |  |  |
| 4. Water Consumption (Less is better) |  |  |  |  |  |  |  |  |  |
| 5. Soil Disturbance (Less is better) |  |  |  |  |  |  |  |  |  |
| 6. Disease Resistance |  |  |  |  |  |  |  |  |  |
| 7. Ease of Operation |  |  |  |  |  |  |  |  |  |
| 8. Nutrient Optimization |  |  |  |  |  |  |  |  |  |
| 9. Crop Diversification Promotion |  |  |  |  |  |  |  |  |  |

**Part 3: Criteria Weight Assignment**

Please assign relative importance to each criterion on a 5-point linguistic scale.

| Criterion | Importance (Select One) |
| --- | --- |
| Affordability | **VG / G / M / P / VP** |
| Maximum Yield | **VG / G / M / P / VP** |
| Climate Resilience | **VG / G / M / P / VP** |
| Water Consumption Efficiency | **VG / G / M / P / VP** |
| Soil Disturbance Minimization | **VG / G / M / P / VP** |
| Disease Resistance | **VG / G / M / P / VP** |
| Ease of Operation | **VG / G / M / P / VP** |
| Nutrient Optimization | **VG / G / M / P / VP** |
| Crop Diversification Promotion | **VG / G / M / P / VP** |

**Section B: Details of experts and Parameter Selection**

*B.1 Experts Details*

To achieve a thorough and contextually relevant assessment of the decision criteria, a structured process of expert elicitation was implemented. This process included experts from the field. These individuals were selected based on their acknowledged expertise and varied professional backgrounds, as detailed below:

1. Prof. Joe Magner (Co-author), Research Professor, University of Minnesota; 34 years’ Experience of working at Minnesota Pollution Control Agency
2. Prof. J Adinarayana, IIT Bombay, India
3. Prof. Yashwant Bhaskar Katpatal, VNIT Nagpur, India
4. Prof. John L. Nieber, Professor, University of Minnesota; Past president of the American Institute of Hydrology; 2021 Soil Physics and Hydrology Chair, Soil Society of America
5. Dr. Mark Deutschman, Research Director, International Water Institute
6. Ms. Shannon Carpenter, Natural Resources Conservation Service, State Water Quality Specialist, USDA, Minnesota
7. Chris Lenhart, Assistant Professor, University of Minnesota
8. Brooke Hacker, DNR Clean Water Specialist
9. Beth Fisher, Minnesota State University
10. Karen Gran, fluvial geomorphologist, University of Minnesota
11. Dave Wall, Hydrologist and Research Scientist, Watershed Division, Minnesota Pollution Control Agency

The selection of experts was designed to ensure a well-rounded mix of viewpoints from academia, government, and practical fields. The evaluation utilized the Delphi method, a recognized technique for building consensus, which was carried out over three rounds to reduce personal biases and improve reliability. During the initial round, experts received a detailed briefing document outlining the study's goals, criteria definitions, and the alternatives being considered.

Specialists were tasked with evaluating the significance of nine different criteria, such as cost-effectiveness, maximizing yield, and conserving water, by using descriptive terms like Very Low, Low, Medium, High, and Very High. These qualitative assessments were then systematically transformed into triangular fuzzy numbers for computational analysis.

Tables 2 and 3 display the final normalized weights, which reflect a consensus reached by the expert panel. These weights were subsequently incorporated into the fuzzy extent analysis framework to assess and rank irrigation and tillage options based on the established criteria. The thorough and transparent approach, grounded in expert input, enhances the validity of the prioritization process and bolsters the overall credibility of the results.

*B.2 Detailed criteria for parameter selection*

A thorough review of the literature was carried out to examine the criteria for decision-making in sustainable agriculture, irrigation, and tillage methods. Recent influential studies, such as those by Aula et al. (2023), Kumar et al. (2023), and Wu et al. (2023), have emphasized the common indicators used to assess both agronomic and environmental performance.

These studies frequently cited parameters such as yield potential, water saving capacity, and resilience to climate variability as critical.

The Delphi method was utilized, engaging 10 senior experts from academia, government, and practical implementation. Over three rounds of feedback, they:

- Reviewed a preliminary list of ~15 criteria drawn from the literature.
- Rated each based on its practical relevance, measurability, and impact on sustainability.
- Narrowed down to nine core parameters based on consensus.

Specialists also made sure that the chosen parameters correspond with the practical decision-making requirements, especially for small-scale farmers and local organizations.

**Delphi Process**:

- Conducted in three iterative rounds to reach consensus and reduce bias.
- A preliminary set of criteria, based on the latest research concerning sustainable agriculture and irrigation systems, was given to experts.
- To encourage a consensus of views, feedback was made anonymous and reassessed in later stages.

**Finalized Evaluation Parameters** (selected based on relevance, applicability, and consensus):

1. **Affordability** – Economic feasibility of adoption for small and marginal farmers.
2. **Max Yield** – Impact on crop productivity and return per hectare.
3. **Climate Resilience** – Ability to withstand climatic extremes (e.g., drought, excess rainfall).
4. **Water Use Efficiency** – Optimization and conservation of irrigation water.
5. **Less Soil Disturbance** – Preservation of soil structure and reduction in compaction.
6. **Disease Resistance** – Influence on reducing crop susceptibility to pests and diseases.
7. **Ease of Operation** – Labor requirement, mechanization needs, and practical usability.
8. **Optimized Nutrient Management** – Enhancement of nutrient uptake and minimization of leaching or volatilization losses.
9. **Promotion of Crop Diversification** – Compatibility with multiple cropping systems and crop rotation flexibility.

**Section C: Detailed calculations of the method used**

*C.1 Calculation of weights*

To evaluate irrigation practices against sustainability-oriented criteria, we employed a fuzzy extent analysis approach for weighting. A panel of five experts rated the importance of nine evaluation criteria: Affordability, Maximum Yield, Climate Resilience, Water Less Consumption, Less Soil Disturbance, Disease Resistance, Easy Operation, Optimized Nutrient Use, and Promoting Crop Diversification. Each expert used a linguistic scale—Very Good (VG), Good (G), Moderate (M), Poor (P), and Very Poor (VP)—to assess the significance of each criterion.

These linguistic ratings were converted into Triangular Fuzzy Numbers (TFNs) based on the following scale:

Table C.1: Linguistic terms corresponding to Triangular fuzzy numbers

| **Linguistic Term** | **TFN (l, m, u)** |
| --- | --- |
| Very Poor (VP) | (0.0, 0.0, 0.25) |
| Poor (P) | (0.0, 0.25, 0.5) |
| Moderate (M) | (0.25, 0.5, 0.75) |
| Good (G) | (0.5, 0.75, 1.0) |
| Very Good (VG) | (0.75, 1.0, 1.0) |

The TFNs across five experts for each criterion were aggregated using the arithmetic mean, and the resulting fuzzy numbers were defuzzified using the Center of Gravity (COG) method:

$$Crisp weight=\frac{l+u+m}{3}$$

Table C.2: Final Weights after Calculation

| **Criterion** | **Final Normalized Weight** |
| --- | --- |
| Affordability | 0.180 |
| Maximum Yield | 0.168 |
| Climate Resilience | 0.165 |
| Water Less Consumption | 0.120 |
| Less Soil Disturbance | 0.095 |
| Disease Resistance | 0.085 |
| Easy Operation | 0.078 |
| Optimized Nutrient Use | 0.063 |
| Promoting Crop Diversification | 0.046 |

*C.2 Fuzzy Synthetic extent*

**Step 1:** Assignment of Triangular Fuzzy Numbers (TFNs) Based on Expert Ratings

Each irrigation practice was evaluated across nine decision criteria using a linguistic scale (Very Poor to Very Good). These qualitative inputs were then mapped to Triangular Fuzzy Numbers (TFNs), where each rating is represented as a triplet (l, m, u) corresponding to the lower, middle, and upper bounds of expert judgment.

The TFNs for this specific irrigation practice (e.g., Surface Irrigation with Mulch – SIM) across the criteria are as follows:

Table C.3: l,m,u format for Sprinkler Irrigtion (SI)

| **Criteria** | **SI_L** | **SI_M** | **SI_U** |
| --- | --- | --- | --- |
| Affordable | 0.3 | 0.5 | 0.7 |
| Max Yield | 0.5 | 0.7 | 0.9 |
| Climate Resilient | 0.3 | 0.5 | 0.7 |
| Water Less Consumption | 0.5 | 0.7 | 0.9 |
| Less Soil Disturbed | 0.3 | 0.5 | 0.7 |
| Disease Resistance | 0.3 | 0.5 | 0.7 |
| Easy Operation | 0.3 | 0.5 | 0.7 |
| Optimized Nutrient | 0.3 | 0.5 | 0.7 |
| Promoting Crop Diversification | 0.3 | 0.5 | 0.7 |

Similarly, it would be done for all the other Irrigation practices where the TFNs would be written in l,m,u format

**Step 2:** Calculating $S_{i}$ using equation (1).

$S_{i}=\frac{\sum_{j=1}^{n} M_{ij}}{\sum_{i=1}^{m} \sum_{j=1}^{n} M_{ij}}$

$\sum_{j=1}^{n} M_{ij}$ : Aggregation of all fuzzy values for alternative $i$ across $n$ criteria.

$\sum_{i=1}^{m} \sum_{j=1}^{n} M_{ij}$ : Total aggregation of all fuzzy values across all alternatives and criteria.

For a group of TFNs, the addition formula illustrates that the fuzzy sum is calculated by independently adding the corresponding lower, middle, and upper values of all the alternatives. Eqn(2)

For instance:

$$Sum\_l=SI\_l+DI\_l+MI\_l+Alt.Fi\_l+PRD\_l+GPI\_l+SIM\_l+CFI\_L$$

$$Sum\_l=0.3+0.3+0.3+0.5+0.3+0.5+0.3+0.5+0.5=3.5$$

Similarly, $Sum\_m and Sum\_u$ is also calculated for all the parameters and criteria. The process of dividing TFNs is carried out using Equation (3)

Then calculating Fuzzy synthetic extent by equation 1,

$S_{1}=\frac{0.3}{3.5}{,S}_{2}=\frac{0.3}{3.5}$, $S_{3}=\frac{0.3}{3.5}$…………

The Fuzzy synthetic extent of SI is represented in table C.3

Table C.3 Fuzzy synthetic analysis for Sprinkler Irrigation (SI)

| **Criteria** | **SI_L** | **SI_M** | **SI_U** |
| --- | --- | --- | --- |
| Affordable | 0.0857 | 0.09433 | 0.09859 |
| Max Yield | 0.1428 | 0.13207 | 0.12857 |
| Climate Resilient | 0.09677 | 0.10204 | 0.10606 |
| Water Less Consumption | 0.1162 | 0.11475 | 0.11538 |
| Less Soil Disturbed | 0.07692 | 0.08771 | 0.09333 |
| Disease Resistance | 0.1034 | 0.10638 | 0.10769 |
| Easy Operation | 0.09677 | 0.10204 | 0.10447 |
| Optimized Nutrient | 0.090909 | 0.09803 | 0.10294 |
| Promoting Crop Diversification | 0.12 | 0.1162 | 0.11475 |

*C.3 Degree of Possibility Matrix.*

Equation 2 is used to further compute the degree of possibility matrix, utilizing values from Table 6 related to irrigation practices.

Step-by-Step Calculation of Degree of Possibility

$$V\left( M_{2}\geq M_{1} \right)$$

Step 1: Input Triangular Fuzzy Numbers (TFNs) for Affordability

Let’s take example fuzzy values from Table 6:

- Sprinkler Irrigation (SI):

$$M_{1}=\left( l_{1},m_{1},u_{1} \right)=\left( 0.3,0.5,0.7 \right)$$

- Drip Irrigation (DI):

$$M_{2}=\left( l_{2},m_{2},u_{3} \right)=\left( 0.7,0.8,0.9 \right)$$

Step 2: Compare the Fuzzy Values Eqn (4)

$$V\left( S_{i}\geq S_{j} \right)= \left\{ \begin{aligned} 1, if m_{i}\geq m_{j} \\ \frac{l_{i}-u_{i}}{\left( m_{i}-u_{i} \right)+\left( m_{j}-l_{j} \right)}, \\ 0, if u_{i} < l_{j} \end{aligned} \right.if u_{i}\geq l_{j} and m_{i}< m_{j}$$

Step 3: Apply the Rule

We observe:

$$u_{1}=0.7 \& l_{2}=0.7$$

Here:

$${m_{2}>m}_{1} (ie, 0.8>0.5)$$

$$l_{2}=u_{1}=0.7$$

So, we fall in the second case

Now apply the formula:

$$V(M2\geq M1)=\frac{l_{2}-u_{1}}{\left( m_{1}-u_{1} \right)-(m_{2}-l_{2})}$$

$$\frac{0.7-0.7}{(0.5-0.7)-(0.8-0.7)}$$

$$=0$$

However, degree of possibility can't be negative or undefined. Since the numerator is zero and denominator is negative, this means:

$$V\left( M_{2}\geq M_{1} \right)=0$$

$$V\left( M_{DI}\geq M_{SI} \right)=0$$

This means that in terms of affordability, Drip Irrigation (DI) is not more preferable than Sprinkler Irrigation (SI) under this specific fuzzy scenario.

Similarly all other matrix are created with respect to each irrigation practice and for tillage practice , example given in table 7 and 8 of manuscript.

*C.3 Ranking of the Alternatives*

For ranking the irrigation practices of table 8 and 7 the normlised score and normalised weighted score were calculated

*C3.1 Normalized Score (NS)*

$$Normalized Score \left( NS \right)=\frac{Total Sum of All Preferences}{Total sum of all the preferences}$$

In this case:

- **Sum for each alternative** = count of how many times it dominates others (value = 1).
- **Total sum across all alternatives** = 54 (as given in table 8).

Example for Sprinkler Irrigation (SI):

$$\left( NS_{SI} \right)=\frac{5}{54}=0.092592593$$

*C3.2 Normalized Weighted Score (NWS)*

$$Normalized Weighted Score \left( NWS \right)=Normalized Score\times Weight$$

Where:

Weight = 0.18 (same for all alternatives in your example).

So for SI:

$$\left( NWS_{SI} \right)=0.092592593\times0.18=0.0166666667$$

*C3.3 Ranking of individual Rank*

Sort alternatives in descending order of Normalized Weighted Score. The higher the NWS, the better the rank (Rank 1 = best).

*C3.4 Overall ranking of the Irrigation and tillage practices*

*Final Aggregated Score*

The Final Aggregated Score for each irrigation alternative is calculated using simple weighted summation of normalized weighted scores across all criteria.

$$Final Aggregated Score=\sum_{i=1}^{n} S_{i}$$

Where, $S_{i}$ = Normalized Weighted Score of the alternative for the **i-th criterion**

$$n=9$$

For example, (Sprinkler Irrigation - SI):

$$Final Aggregated Score S=0.016666667+0.017777778+0.008875+0.014444444$$

$$+0.003703704+0.010769231+0.006153846+0.00808511+0.008727273\boldsymbol{=0.091926}$$

In similar way for each criterion, the final combined score is determined for all parameters related to irrigation and tillage practices, with the ultimate rankings presented in Tables 9 and 10, respectively.
